# Insights into the Pharmacokinetics and In Vitro Cell-Based Studies of the Imidazoline I_2_ Receptor Ligand B06

**DOI:** 10.3390/ijms23105408

**Published:** 2022-05-12

**Authors:** Andrea Bagán, José A. Morales-García, Christian Griñán-Ferré, Caridad Díaz, José Pérez del Palacio, Maria C. Ramos, Francisca Vicente, Belén Pérez, José Brea, María Isabel Loza, Mercè Pallàs, Carmen Escolano

**Affiliations:** 1 Laboratory of Medicinal Chemistry (Associated Unit to CSIC), Department of Pharmacology, Toxicology and Medicinal Chemistry, Faculty of Pharmacy and Food Sciences, Institute of Biomedicine (IBUB), University of Barcelona, 08028 Barcelona, Spain; abaganpolonio@ub.edu; 2 The Network Center for Biomedical Research in Neurodegenerative Diseases (CIBERNED), Department of Cell Biology, School of Medicine, Complutense University (UCM), 28040 Madrid, Spain; jmoral06@ucm.es; 3 Pharmacology Section, Department of Pharmacology, Toxicology and Medicinal Chemistry, Faculty of Pharmacy and Food Sciences, Institut de Neurociències, University of Barcelona, 08028 Barcelona, Spain; christian.grinan@ub.edu (C.G.-F.); pallas@ub.edu (M.P.); 4 Fundación MEDINA, Centro de Excelencia en Investigación de Medicamentos Innovadores en Andalucía, Avda. del Conocimiento 34, 18016 Armilla, Spain; caridad.diaz@medinaandalucia.es (C.D.); jose.perezdelpalacio@medinaandalucia.es (J.P.d.P.); carmen.ramos@medinaandalucia.es (M.C.R.); francisca.vicente@medinaandalucia.es (F.V.); 5 Department of Pharmacology, Therapeutic and Toxicology, Autonomous University of Barcelona, 08193 Barcelona, Spain; belen.perez@uab.cat; 6 Innopharma Screening Platform, BioFarma Research Group, Centro de Investigación en Medicina Molecular y Enfermedades Crónicas (CIMUS), Universidad de Santiago de Compostela, 15706 Santiago de Compostela, Spain; pepo.brea@usc.es (J.B.); mabel.loza@usc.es (M.I.L.)

**Keywords:** imidazoline I_2_ receptor ligand, pharmacokinetics, bicyclic α-iminophosphonate, metabolic profile, neuroprotection, Alzheimer’s disease, Parkinson’s disease

## Abstract

The impact of neurodegenerative diseases (ND) is becoming unbearable for humankind due to their vast prevalence and the lack of efficacious treatments. In this scenario, we focused on imidazoline I_2_ receptors (I_2_-IR) that are widely distributed in the brain and are altered in patients with brain disorders. We took the challenge of modulating I_2_-IR by developing structurally new molecules, in particular, a family of bicyclic α-iminophosphonates, endowed with high affinity and selectivity to these receptors. Treatment of two murine models, one for age-related cognitive decline and the other for Alzheimer’s disease (AD), with representative compound **B06** ameliorated their cognitive impairment and improved their behavioural condition. Furthermore, **B06** revealed beneficial in vitro ADME-Tox properties. The pharmacokinetics (PK) and metabolic profile are reported to de-risk **B06** for progressing in the preclinical development. To further characterize the pharmacological properties of **B06**, we assessed its neuroprotective properties and beneficial effect in an in vitro model of Parkinson’s disease (PD). **B06** rescued the human dopaminergic cell line SH-SY5Y from death after treatment with 6-hydroxydopamine (6-OHDA) and showed a crucial anti-inflammatory effect in a cellular model of neuroinflammation. This research reveals **B06** as a putative candidate for advancing in the difficult path of drug discovery and supports the modulation of I_2_-IR as a fresh approach for the therapy of ND.

## 1. Introduction

**Figure 1:**
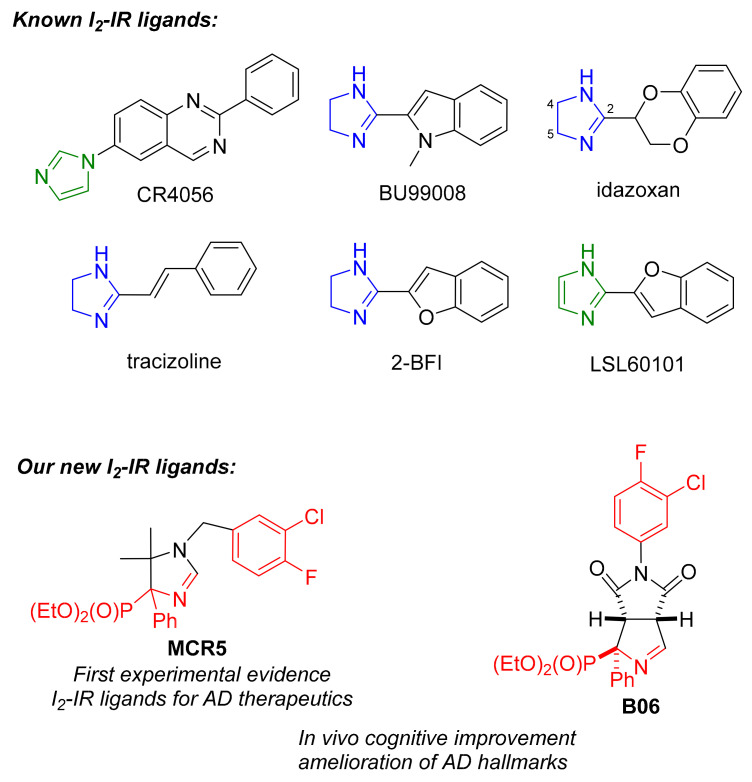
Structure of the known I_2_-IR ligands (2-imidazoline nucleus in blue and imidazole nucleus in green) and our representative new I_2_-IR ligands, **MCR5** and **B06**.

Imidazoline I_2_ receptors (I_2_-IR) are nonadrenergic binding sites recognized by the radioligands [^3^H]-idazoxan and [^3^H]-*p*-aminoclonidine, as well as and with the lowest affinity, by [^3^H]-clonidine [1,2,3]. I_2_-IR are found in the central and peripheral nervous system and many organs and tissues [4,5]. The alteration of I_2_-IR is a hallmark in a plethora of neurodegenerative illnesses, such as brain disorders, including Alzheimer’s disease (AD), Parkinson’s disease (PD), Huntington’s disease, depression, and glial tumours [6,7,8,9,10,11,12,13]. Compounds with a high affinity for I_2_-IR modulate many physiological and pathological processes such as analgesia and inflammation [14,15]. Two I_2_-IR ligands, CR4056 and [^13^C]BU99008, are progressing in clinical trials, unveiling the relevance of these receptors in therapeutics. The analgesic CR4056, described as the first-in-class I_2_-IR ligand, is in phase II for osteoarthritis and postoperative dental pain, and [^13^C]BU99008 is in phase I for PET diagnosis in patients suffering from AD (Figure 1) [16,17,18].

Despite the potential of I_2_-IR in therapeutics, their complete pharmacological characterization is still in its infancy due to their high heterogeneous nature and the absence of structural details. The disclosure of their pharmacological role relies on their modulation by I_2_-IR ligands and the observation of the physiological responses. Known I_2_-IR ligands are scarce and with a limited structural pattern based on the 4,5-nonsubstituted-2-heterocyclic-2-imidazoline nucleus (e.g., idazoxan, tracizoline, 2-BFI), with the only exceptions of CR4056 and LSL60101 endowing an imidazole motif (Figure 1) [19,20,21]. In this context, we have contributed by increasing the arsenal of ligands with structurally unprecedented new families that showed outstanding affinities to I_2_-IR devoid of α_2_-adrenoceptors (α_2_-AR) in human brain tissues.

Therefore, in 2017, we reported the synthesis and pharmacological characterization of a family of (2-imidazolin-4-yl)phosphonates that showed excellent affinity to I_2_-IR [22,23]. Considering that I_2_-IR are increased in the patients that suffered from AD [24], we explored the implications of a representative member of this family, the [1-(3-chloro-4-fluorobenzyl)-5,5-dimethyl-4-phenyl-4,5-dihydro-1*H*-imidazol-4-yl]phosphonate, named as **MCR5**, in neurodegenerative diseases (ND). Our concern in this field is due to the huge worldwide impact of ND, mainly AD [25] and PD [26]. The WHO estimates that more than 50 million people are suffering from AD and about 7 million from PD. Unfortunately, these numbers are dramatically increasing every year. Aging of the population and the lack of effective treatments place AD as one of the most serious unmet medical needs that humankind must face in the upcoming years [27]. Avoiding the collapse of health systems and ameliorating the suffering of patients and families guide the scientific community efforts. As no new cognitive enhancer drug has reached the market in nearly two decades, identifying new therapeutic targets and entities is a priority for medicinal chemistry programs [28]. Accordingly, we treated with **MCR5** a murine model of age-related cognitive decline, the senescence-accelerated mouse prone 8 (SAMP8), resulting in a cognitive improvement and amelioration of hallmarks. This study constituted the first experimental evidence that demonstrated the potential of the modulation of I_2_-IR as a new therapy for AD [29]. Additionally, improvement in the behavioural and psychological symptoms of dementia, including fear–anxiety, depressive-like behaviour, and memory decline, present in most ND, were observed when treating an orally SAMP8 murine model with **MCR5** [30]. There are a few studies that discuss the presence of the I_2_-IR in post-mortem tissue from PD patients, but none describe its role in the development of PD [7].

Driven by the excellent precedented pharmacological results and the possibility of opening new structural opportunities, we reported a family of bicyclic α-iminophosphonates synthetised by a [3+2]cycloaddition reaction [31]. We selected representative diethyl (1*RS*,3a*SR*,6a*SR*)-5-(3-chloro-4-fluorophenyl)-4,6-dioxo-1-phenyl-1,3a,4,5,6,6a-hexahydropyrrolo[3,4-*c*]pyrrole-1-phosphonate, named as **B06**, that shares an α-phenyl-α-iminophosphonate function and a 3-chloro-4-fluorophenyl group with **MCR5** (highlighted in red in Figure 1), and it is endowed with high affinity and selectivity to I_2_-IR. Structurally, **B06** does not fit into the restricted substitution pattern of I_2_-IR ligands, with its bicyclic α-iminophosphonate structure being the first described non-imidazoline/non-imidazole-containing compound, showing outstanding affinity to I_2_-IR. **B06** displays in the membranes of the post mortem human frontal cortex, a brain area that shows an important density of I_2_-IR and α_2_-AR, a pK*i* = 8.56 ± 0.32, and a ratio of I_2_/α_2_ = 195 in competitive binding studies against the selective I_2_-IR radioligand [^3^H]2-BFI and the selective α_2_-AR radioligand [^3^H]RX821002. Note that the standard idazoxan showed a lower pK*i* than **B06** with a value of 7.41 ± 0.63 and non I_2_/α_2_ selectivity. Considering the localization of I_2_-IR in the central nervous system (CNS), the good ability of **B06** to cross the blood−brain barrier (BBB) was an essential requirement with a Pe value in a parallel artificial membrane permeability assay (PAMPA) well above the threshold established for high BBB permeation. **B06** was devoid of cytotoxicity in a plethora of culture cells. In silico studies offered confidence for performing in vitro assays that showed non-warnings in the physicochemical properties (solubility and chemical stability), microsomal stability, cytochromes inhibition, hERG inhibition, and plasma protein binding. A receptor characterization panel de-risked our representative compound evaluated in vivo, exhibiting hypothermic properties and neuroprotection in the murine model of age-related cognitive decline, the SAMP8, as well as in a murine model of AD, the 5xFAD [32,33]. Thus, **B06** exerts a beneficial effect by improving cognition and ameliorating anxiety-like behaviour in the SAMP8 murine model. Modulation of I_2_-IR by **B06** reduces neuroinflammation, oxidative stress and calcineurin protein levels in SAMP8 [34].

**Figure 2:**
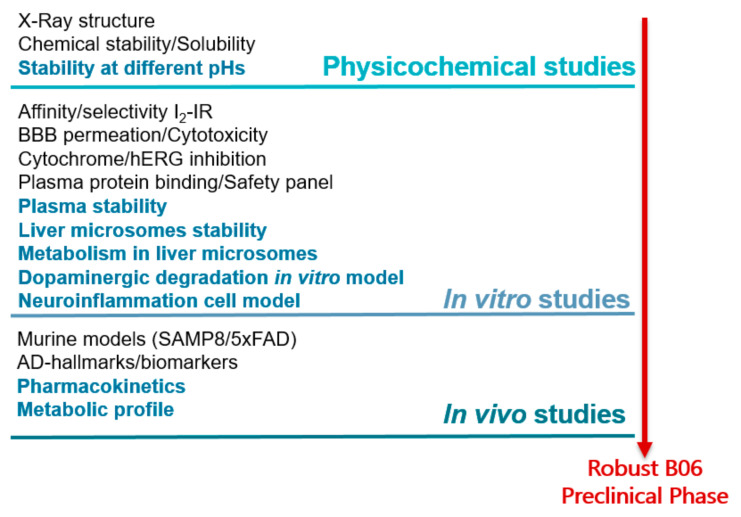
Summary of the previous (black) and current (blue) studies of **B06**.

To progress the promising candidate **B06** in preclinical studies, here, we report parameters to be assessed in the early discovery process, such as physicochemical characteristics (chemical stability at different pH) and complete ADME parameters, such as plasma stability. To shed light on the biotransformation of **B06**, we undertook studies considering the stability in mouse and human liver microsomes, pharmacokinetics (PK), in vitro and in vivo metabolic profile, and the identification of metabolites of **B06**. To better describe the pharmacological features of **B06** and considering the paucity of information on the role of I_2_-IR ligands in PD, we used a model of dopaminergic neurodegeneration to evaluate how **B06** could protect neurons. Furthermore, since most ND such as AD and PD are characterized by the presence of an exacerbated and sustained neuroinflammatory process, which is partly responsible for the development of the disease, we have completed the study of the in vitro biological activity of **B06** using a cellular model of neuroinflammation (Figure 2).

Overall, this study boosts **B06** as a promising candidate for further preclinical development in AD indication or other ND diseases. The in-depth characterization of **B06** will contribute to build a robust comprehensive understanding of the pharmacological implications of I_2_-IR and their ligands in therapeutics.

## 2. Results and Discussion

### 2.1. Solubility and Chemical Stability of **B06** at Different pH

The solubility of a compound is an essential physicochemical property that drives drug absorption and optimizes its therapeutic exposure in plasma and the therapeutic target. The capabilities of the compound to be solved in different media will avoid misunderstanding in the pharmacological results. **B06** showed good solubility in several media, methanol, acetonitrile, and water, and an excellent solubility of 92 µM was found in 1% DMSO and 99% PBS buffer [33]. Furthermore, the chemical stability of **B06** in buffers at different pH was undertaken to measure the degradation by non-enzymatic processes (most common hydrolysis and oxidation). The pH value of the gastrointestinal tract varies from acidic in the stomach to basic in the small and large intestine, and alterations of representative compounds need to be assessed. To this end, a test solution of **B06** (1 μM, 0.1% final DMSO concentration) was incubated with buffer pH 2, 5 and 7.4 at 37 °C. Serial samples were taken at 0, 5, 15, 30, 60 and 120 min. All samples were added immediately to three volumes of methanol containing internal standard in a microtiter plate cooled to halt chemical degradation. All the samples were analysed by LC-MS/MS. The percentage of parent compound remaining at each time point relative to the 0 min sample was calculated from peak area ratios. The chemical stability assay returned the per cent parent compound remaining at each time point for **B06**.

**Figure 3:**
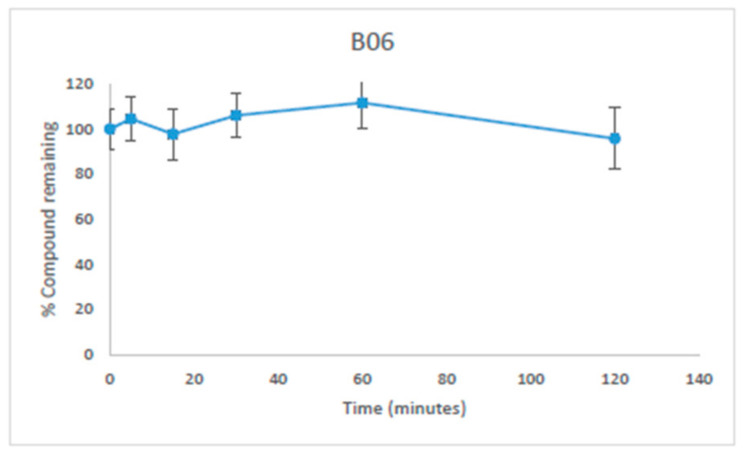
Chemical stability of **B06** at pH = 2.

**Figure 4:**
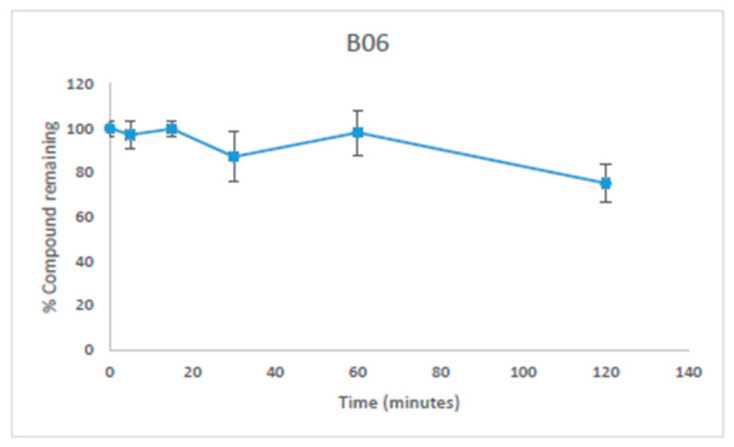
Chemical stability of **B06** at pH = 5.

**Figure 5:**
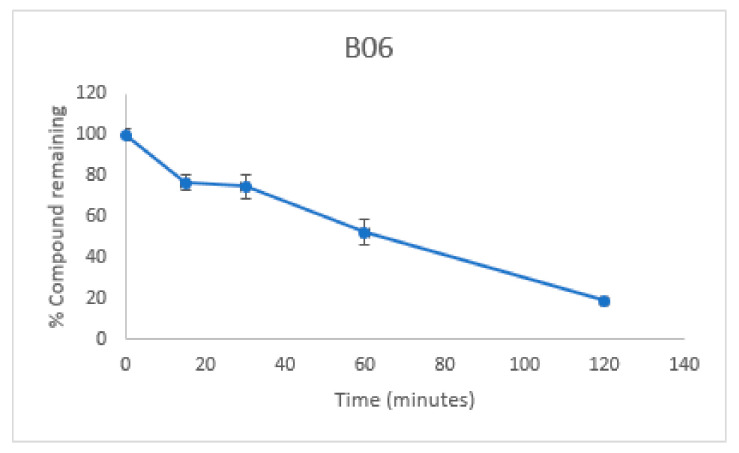
Chemical stability of **B06** at pH = 7.4.

The study of the graphics reveals that **B06** was stable at pH 2, without significant changes in the structure up to 120 min (Figure 3, Table 1). **B06** in a pH 5 media proved to be stable up to 1 h, and 25% of the compound suffered structural changes during the next hour (Figure 4, Table 2). In neutral media (pH = 7.4), **B06** remained unchanged during the first minute, and at 15 min, 25% of the compound was transformed. At 30 min and pH = 7.4, 75% of **B06** was detectable, with half the amount at 60 min, and only 19% at 2 h (Figure 5, Table 3). This study gave us an overview of the chemical behaviour of **B06**, depending on the pH of the media versus time.

### 2.2. Plasma Stability of **B06** at Mouse Plasma

Stability in liver microsomes does not imply stability in plasma, where many hydrolytic enzymes are circulating. In previous studies, the mice microsomal percentage remanent of **B06** at 60 min was 65.08, with a t ½ of 91.72 min [33]. The assessment of the plasma stability avoids misleading in vitro data and complex interpretation of the in vivo PK. To this end, 10 µM **B06** in plasma mouse at 37 °C was incubated at different times (0, 60, 180 and 360 min), and acetonitrile was added for precipitating plasma protein. Then, centrifugation led to a supernatant that was analysed for sample quantification. The remaining percentage for **B06** was 100% at 0 and 10 min, 69.7% at 20 min, 48.3% at 40 min and 21.6% at 60 min. Although **B06** displays transformation with time, the percentage of non-modified compounds enables its in vivo activities.

### 2.3. Metabolic Stability of **B06** in Liver Microsomes

In vitro liver microsome metabolite identification studies are essential models for assessing metabolic profiling in early drug discovery. As the compound **B06** progresses through the discovery–development–clinical continuum, the goals of the metabolite analysis may alter, as will the detail with which the experiment is conducted. Early in drug discovery, the compounds being synthesized frequently have poor PK characteristics; thereby, in vitro studies are performed using liver microsomes or hepatocytes as a model to assess the metabolic profiling of the compound.

There are several reasons as to why understanding the metabolic profile of a compound is important. First, the FDA Guidance for Safety Testing of Drug Metabolites [35] recommends in vitro evaluation of interspecies differences in drug metabolism between humans and the animals expected to be used in preclinical safety assessments. If a metabolite is formed only in humans and it is absent in the animal test species, or if the metabolite is present at disproportionately higher levels in humans than in the animal species, then it may be necessary to assess the preclinical safety of the metabolite in question. Second, understanding the metabolic liability of a compound is important in directing chemistry. If a compound is rapidly cleared, then identifying which functional groups are undergoing metabolism will be valuable in understanding how the chemistry may be altered to reduce compound degradation.

#### 2.3.1. Metabolic Stability of **B06** in Human Liver Microsomes

The metabolic stability using human liver microsomes (HLM) is directly related to the elimination of the drug from the body and significantly affects efficacy and safety. In the assay, the reaction was initiated by adding HLM (1 mg/mL) to an equal volume of buffer solution containing the test compound and cofactors at the proper concentrations. Reactions without NADPH were also incubated to rule out non-NADPH metabolism or chemical instability in the incubation buffer. Verapamil was included as a positive control to monitor incubation course. All reactions were terminated using 60 µL of ice-cold acetonitrile at 0, 5, 15, 30, 45, and 60 min. The plates were centrifugated at 3500 rpm for 15 min. All experiments were conducted in triplicate. Samples were monitored for **B06** disappearance by LC-MS in MRM mode according to a quantitation method for **B06**. The percentage of the remaining parent compound and the time course of metabolic stability for verapamil, positive control, and **B06** at each incubation time are displayed in Table S2 and Figure S1 and Table 4 and Figure S2, respectively.

The analysis of the percentage of **B06** remaining in minus NADPH cofactor incubations did not show the metabolism of the parent from nondependent NADPH enzymes. The present study indicates that **B06** displays a half-life value equal to 28.4 min (Table S3). Consequently, the predicted intrinsic clearance calculated for **B06** is 23.5 mL/min/mg protein (Table S3). Attending to the classification bands typically used for categorizing compounds into low, medium or high clearance (Table S4), **B06** showed hepatic clearance classified as medium clearance.

#### 2.3.2. Metabolic Stability of **B06** in Mouse Liver Microsomes

Metabolic stability of **B06** in mouse liver microsomes (MLM) was carried out as in the previous section, but instead of using HLM, the study required MLM.

The percentages of the remaining parent compound and the time course of metabolic stability for verapamil, positive control, and **B06** at each incubation time are displayed in Table S6 and Figure S3 and Table 5 and Figure S4, respectively. **B06** showed a half-life value equal to 16.23 min (Table S7). The intrinsic clearance calculated for **B06** was 153.7 mL/min/mg protein, being classified as high clearance (Table S8).

Note that the half-life value and the intrinsic clearance for **B06** outcomes discussed above are in the same range as in liver microsomes of both species, mice and humans.

### 2.4. Metabolic Profiling of **B06** in Liver Microsomes

The objective of the study is to evaluate the formation of the main metabolites from **B06** in HLM incubations and MLM incubations preceding the in vivo metabolic study, using these same incubations to identify the possible metabolites. The assay was initiated by adding HLM solution (1 mg/mL) or MLM solution (1 mg/mL) to an equal volume of buffer solution containing **B06** and cofactors. Reactions without NADPH were also incubated to rule out non-NADPH metabolism or chemical instability in the incubation buffer. Verapamil was included as positive control to monitor incubation course. All reactions were terminated using 60 µL of ice-cold acetonitrile at 0, 5, 15, 30, 45, and 60 min. The plates were centrifugated at 3500 rpm for 15 min. All experiments were conducted in triplicates. Samples were analysed by LC-HMRS in positive ionization mode. Compound incubations without NADPH and compound reference standard were used as interference control for each incubation sample.

#### 2.4.1. Metabolic Profiling of **B06** in Human Liver Microsomes

**Scheme 1:**
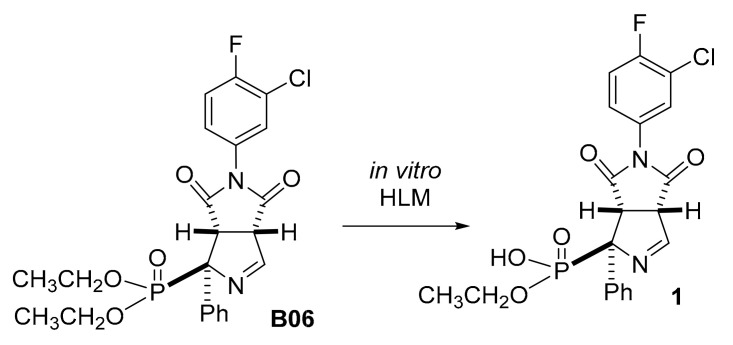
Proposed product **1** resulting from the in vitro HLM assay.

The potential metabolite **1**, ethyl hydrogen [(1R,3aS,6aS)-5-(3-chloro-4-fluorophenyl)-4,6-dioxo-1-phenyl-1,3a,4,5,6,6a-hexahydropyrrolo[3,4-c]pyrrol-1-yl]phosphonate, was tentatively identified in HLM incubations from 15 to 60 min. The assigned molecular formula was C_20_H_17_N_2_O_5_FPCl, resulting in the loss of C_2_H_4_ (Scheme 1). The transformation can be accounted for in the hydrolysis, in a Phase I metabolic conversion, of the ethoxy group of the phophonate function. The hydrolysis of the phosphonate ester group to the corresponding ethyl hydrogen phosphonate derivative is a plausible reaction in the physiological aqueous media (Scheme 1). In the metabolite chromatogram at 15 min, the metabolite detected was 8.4%, at 30 min was 17.8%, at 45 min was 26.2% and at 60 min was 36% of the area (page S5, Table S9).

**Figure 6:**
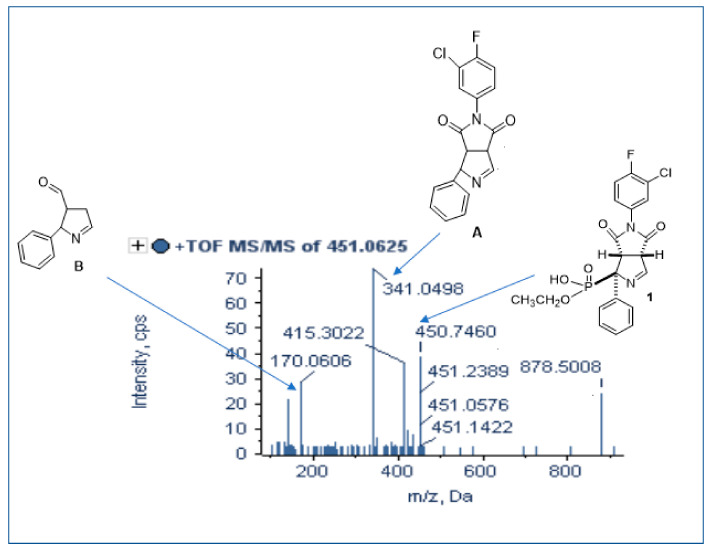
MS/MS spectra at 15 min of the characteristic fragmentation pattern of **B06** metabolite 1 in HLM.

From the TOF MS/MS spectra at 15 min, several peaks were selected for assignment. The signal with the mass of the product **B06** without 25 units, 451.2389, could involve the loss of a C_2_H moiety. Other peaks selected for the assignment were 341.0498 that could be the fragment **A** without the diethylphosphonate ester of **B06**, and 170.0606 that could correspond to the fragment **B** with a 3,4-dihydro-2H-pyrrole nucleus (Figure 6).

#### 2.4.2. Metabolic Profiling of **B06** in Mouse Liver Microsomes

The objective of the study is to evaluate the formation of the main metabolites from **B06** in MLM incubations.

Compound **1**, identified in the metabolic profile of **B06** in HLM, was tentatively detected in MLM incubations from 5 to 60 min. In the metabolite chromatogram at 5 min, compound **1** was detected in 10.7%, at 15 min its area was equal to 30.9%, at 30 min its presence increased to 60.9%, at 45 min it was found to be 81.6%, and at 60 min it represented 88.9% of the area (Page S5, Table S10). Note that the half-life value and the intrinsic clearance for **B06** outcomes discussed above are in the same range in HLM and MLM. These results could be a sign of a similar stay time of **B06** in the organism in both animals species.

### 2.5. Synthesis of the Metabolite Identified in the Metabolic Profiling of **B06** in Human Liver Microsomes

Metabolite **1** proposed from the biotransformation via Phase I of **B06** by HLM prompted us to undertake its synthesis for unequivocal identification. To this end, the diethyl phosphonate ester group of **B06** was hydrolysed by treatment with trimethylsilyl bromide in dichloromethane furnishing the desired compound **1**.

The synthesized compound **1,** was compared to the potential metabolite identified in both liver microsome incubations preceding the in vivo metabolic study, showing the same retention time and spectral information. Consequently, in the liver microsomes of both species, mice and humans, a metabolism of phase I occurs through the hydrolysis of the phosphonate ester furnishing **1**.

### 2.6. In Vitro Effects of **B06** in a Preclinical Model of Neurodegeneration

#### 2.6.1. Effects of **B06** on Cell Viability in a Dopaminergic Cell Line

**Figure 7:**
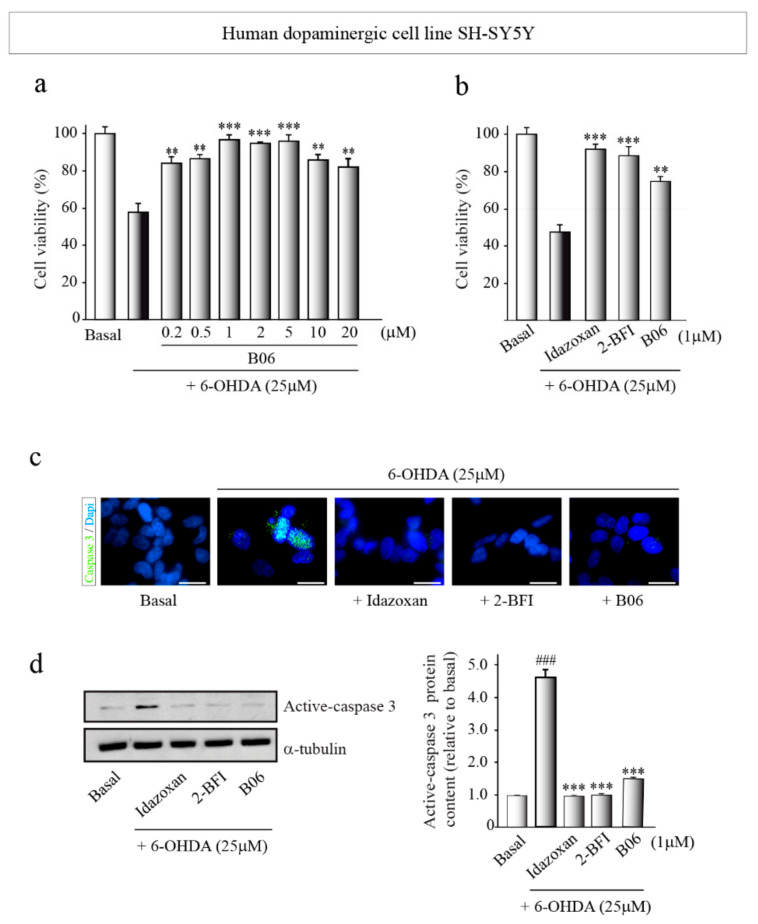
Phenotypic screening of I_2_-IR ligands. Neuroprotective effect of **B06** in vitro. Human dopaminergic cell line SH-SY5Y was exposed to 6-OHDA (25 μM) for 16 h. Cell viability was determined by MTT assay. Values represent the mean ± SD from triplicate determinations repeated at least three times. ** *p* ≤ 0.01, *** *p* ≤ 0.001 versus 6-OHDA-treated cultures. (**a**) Dose– response curve of **B06**. In order to determine the optimal dose of use of **B06** for in vitro analysis, different concentrations of **B06** were used. (**b**) To evaluate the neuroprotective role of **B06**, some cultures were pre-treated with idazoxan, 2-BFI and **B06** (1 μM) for 1 h before 6-OHDA treatment. Apoptotic levels were determined by active caspase 3 (green) immunodetection (**c**) and Western blot quantification (**d**). Representative images of at least three independent experiments are shown. Scale bar, 10 µm. Nuclei were counterstained with DAPI (blue). *** *p* < 0.001 versus 6-OHDA-treated cells. ^###^ *p* < 0.001 versus control (basal) cells.

The process of neurodegeneration results in progressive changes in the structure or function of neurons, leading to devastating neurological conditions characteristic of AD and PD [36]. Due to the lack of information about the role of I_2_-IR ligands in PD development, first, we investigated the neuroprotective effect of **B06** on the human dopaminergic cell line SH-SY5Y. The human neuronal dopaminergic cell line SH-SY5Y has been described as an ideal system for modelling in vitro the characteristics of dopaminergic neurons because they have many features of substantia nigra neurons [37]. We first tested whether the compound per se could have a cytotoxic effect on cell cultures. For this purpose, we treated the cells with different concentrations of the compounds from 200 nM to 20 μM (Page S18, Figure S18). None of the concentrations used were cytotoxic to the cells. Then, we conducted a dose–response experiment in order to select the lowest concentration of **B06** at which the most beneficial effect was obtained (Figure 7a). From this point on, we always use that concentration (1 μM) in our analysis. Now, we tested whether **B06** is able to protect the SH-SY5Y cell line from cell death induced by the toxin 6-hydroxydopamine (6-OHDA), a well-established cell model for dopaminergic neurodegeneration in which our group has extensive experience [38]. For a more in-depth analysis, the level of active caspase 3 was used to determine apoptosis (Figure 7c,d). Qualitatively, our results indicated an increase in the number of SH-SY5Y cells expressing active caspase 3 within 16 h after treatment with 6-OHDA. and this effect was reversed by the treatment with **B06**. A Western blot analysis was used to confirm and quantify these results (Figure 7d). For comparison, two well-known I_2_-IR ligands, idazoxan and 2-BFI, were included as standards (Scheme 1). The results summarized in Figure 7 clearly show that the treatment of SH-SY5Y cells with compound **B06** significantly rescued human dopaminergic cells from 6-OHDA-induced apoptosis. These results are in agreement with previous observations, suggesting that I_2_-IR are dysregulated in patients with ND [6,7,8], providing new data on their role in PD in particular.

#### 2.6.2. Anti-Inflammatory Effects In Vitro of **B06**

**Figure 8:**
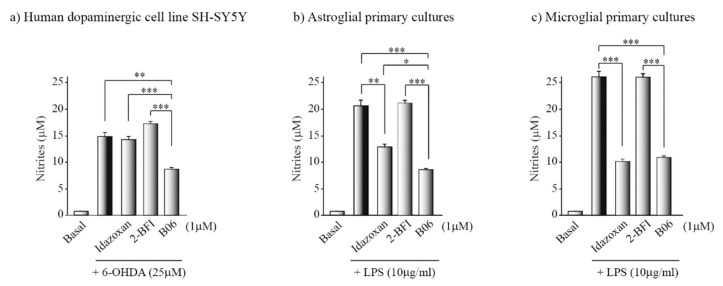
In vitro anti-inflammatory effect of **B06** and comparison with idazoxan and 2-BFI. Nitrite production was measured by the Griess reaction in the supernatant of the SH-SY5Y dopaminergic cell line (**a**), and murine-derived astrocytes (**b**), and microglial primary cultures, (**c**) cells were treated for 16 h with 6-OHDA (25 μM) or for 24 h with lipopolysaccharide (LPS, 10 μg/mL) in the presence of the different compounds (1 μM). Values represent the mean ± SD from triplicate determinations repeated at least three times. * *p* ≤ 0.05, ** *p* ≤ 0.01, *** *p* ≤ 0.001.

Along with the neuronal loss, neuroinflammation is a process related to the onset of the disease, and it is an important contributor to the pathogenesis and progression of these disorders [39]. Since cytotoxicity induced by the neurotoxin 6-OHDA is frequently accompanied by pronounced inflammatory activity, we next evaluated the potential anti-inflammatory effect of **B06** by evaluating the production of nitrites in SH-SY5Y cultures treated with the neurotoxin. In line with the results obtained with the cell viability experiments, the treatment with **B06** significantly reversed the increase in nitrite production elicited by 6-OHDA treatment (Figure 8), suggesting a convincing anti-inflammatory effect of **B06**. Notably, **B06** demonstrated a significantly better anti-inflammatory effect in comparison with other I_2_-IR ligands.

In the CNS, microglia and astrocytes play a key role in regulating inflammatory responses. Moreover, I_2_-IR are widely distributed in the brain and primarily in glial cells [40], suggesting that this receptor plays an important role in neuroinflammation. Therefore, to further evaluate the role of **B06** in inflammatory reactions, we performed experiments in different cell-based assays that mimic in part the neuroinflammatory process in PD [41]. We used primary cultures of astrocytes and microglia treated with bacterial lipopolysaccharide (LPS), a potent inflammatory agent. Then, the potential anti-inflammatory activity of **B06** was tested by evaluating the production of nitrites on primary cultured glial cells. Cultures were incubated with 1 μM of the compound for 1 h, and then cells were treated with LPS for a further 24 h. When primary astrocytes and microglial cells were stimulated with LPS (Figure 8), we observed a significant induction of nitrite production (approximately seven-fold) in the culture medium, an indicator of inflammation. Those cultures previously treated with **B06** showed a significant decrease in nitrite production, showing better results when compared with I_2_-IR ligands idazoxan and 2-BFI, thus confirming the role of **B06** as a potent anti-inflammatory agent. As neuroinflammation is a hallmark of many neurodegenerative diseases, including AD and PD [42], **B06** may have particular relevance for treating disorders that involve inflammation of the nervous system.

### 2.7. In Vivo Pharmacokinetics of **B06**

The physicochemical and biochemical properties of a compound determine its time course after its administration. Parameters such as concentration in bloodstream and tissues, distribution, metabolization, and elimination are fundamental for the description of the pharmacological behaviour and therapeutic characteristics of **B06**. To this end, after the absence of in vitro warnings in the ADME studies and in vivo advantages of **B06**, we proceeded with the PK and metabolic profile studies. Note, due to the therapeutic indication addressed by **B06** in the CNS, we considered the quantity of compounds that rise in brain tissues.

Previously, we reported hypothermia observed in adult rats when treated intraperitoneally with 20 and 35 mg/kg measured 1 and 2 h postinjection and in mice after repeated administration of 20 mg/kg for 5 days of **B06** [33]. It is well stablished that hypothermia causes significant neuroprotection, which also led us to have a wide range of dose confidence for the following studies. Taking into account the absence of toxicity at high doses of **B06**, even at 5 days, and the dose of 5 mg/kg in drinking water during 4 weeks employed in the cognitive studies, we considered performing the PK assays at 10 mg/kg, intraperitoneally.

Thus, two-month-old mice were treated with a single dose of 10 mg/kg intraperitoneally in ten groups (*n* = 3), for consecutive times: 0, 5, 15, 30 min and 1, 2, 4, 6, 8, 24 h. After treatment, mice were euthanized, and the plasma and brain were collected. The experimental groups were composed of three mice, and there was not toxicity in any of the animals. Furthermore, de visu observation after euthanasia showed no toxicity in organs or tissues. These data were in accordance with the previous absence of toxicity at higher doses.

#### 2.7.1. Pharmacokinetics of **B06** in Plasma

**Figure 9:**
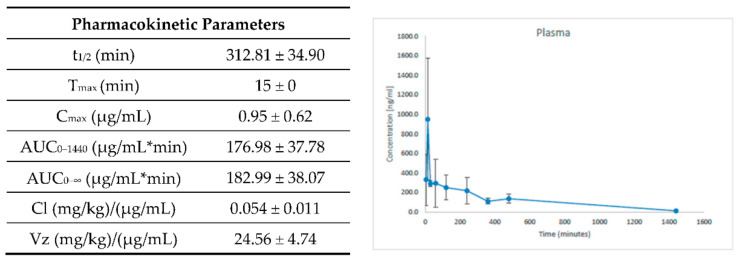
Plasma concentration of **B06** at different times (5 min–24 h) after an intraperitoneal administration of 10 mg/kg. Basic PK parameters were calculated. C_max_: maximum observed concentration, T_max_: time of maximum observed concentration, t_1/2_: terminal elimination half-life, AUC_0–1440_: area under the curve from zero to the last sampling time, AUC_0–∞_: area under the curve from zero extrapolated to infinity, Cl: plasma clearance, Vz: volume of distribution.

The plasma profile and PK parameters of **B06** are shown in Figure 9.

The maximum concentration in plasma (C_max_) was 0.950 ± 0.624 μg/mL and was reached at 15 min (T_max_). Plasma levels, although low, remained fairly consistent for 8 h post-administration and at 24 h drug concentration, were almost undetectable. The half-life time (t_1/2_) was 312.8 min and the AUC_0–1440_/AUC_0–inf_obs_ ratio (0.97) showed that 24 h is a good interval to define the kinetic behaviour of **B06** in plasma after intraperitoneal administration at a dose of 10 mg/kg.

The maximum mean concentration found in plasma does not exceed 1% of the drug administered in a single dose intraperitoneally. Notwithstanding the low levels, the administration of **B06** in drinking water at a dose of 5 mg/kg to the murine models continued at effective levels for 4 weeks. The sustained intake for the animals and the treatment time could account for the beneficial effects of **B06** in the SAMP8 and 5xFAD murine models by improving cognition and ameliorating the neurodegenerative condition. In addition, a reduction in the AD hallmarks was in accordance with the new state of the animals, pointing out the variations in biochemical pathways due to the interaction of **B06** with a therapeutic target.

#### 2.7.2. Pharmacokinetics of **B06** in Brain

**Figure 10:**
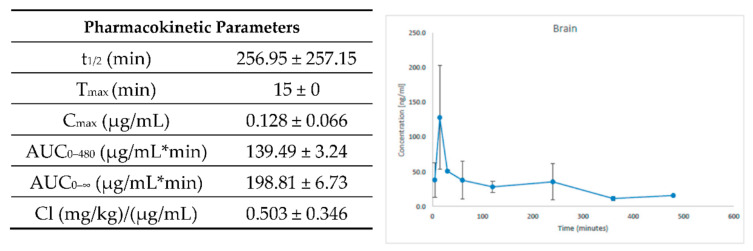
Brain concentration of **B06** at different times (5 min–24 h) after intraperitoneal administration of 10 mg/kg. Basic PK parameters were calculated. C_max_: maximum observed concentration, T_max_: time of maximum observed concentration, t_1/2_: terminal elimination half-life, AUC_0–1440_: area under the curve from zero to the last sampling time, AUC_0–∞_: area under the curve from zero extrapolated to infinity and Cl: the brain clearance.

The quantification in the brain of **B06** and the calculated PK parameters are shown in Figure 10.

The **B06** concentrations found in the brain show that in this organ, the drug profile is identical to that found in plasma, presenting equal T_max_ and similar t_1/2_, 312.81 min in plasma and 256.95 min in the brain. The ratio between brain/plasma concentrations remains constant (approximately 10%). These results indicate the consistent correlation of **B06** levels between plasma and the brain when the drug is administered at a dose of 10 mg/kg.

These data are according to previous PAMPA-BBB results, confirming in vivo the high capacity of the drug to penetrate the BBB.

### 2.8. Metabolic Profiling In Vivo of **B06**

Metabolite identification studies are essential in the early stages of drug discovery and during drug and clinical development and are strongly recommended by the FDA Guidance for Industry on Safety Testing of Drug Metabolites [35]. After performing the liver microsomes studies reported above, we undertook the metabolite determination by seizing the PK study. When entering the body, **B06** will undergo biotransformation via Phase I and Phase II metabolic pathways. In this framework, a study to understand the biotransformation details of **B06** by identifying the main in vivo metabolites from **B06** at different times (30 to 480 min) was performed. The aim was to discard toxic issues and add information on the behaviour underlying beneficial cognitive impairment in the murine model of neurodegeneration and AD after treatment with **B06**. To this end, an aliquot of 50.0 µL mouse plasma and 150.0 µL of cold acetonitrile was added. After vortex mixing for one minute, the samples were centrifuged for 15 min at 13,300 rpm. An aliquot of 110.0 µL of the supernatant was transferred to an auto sampler vial to analyse by LC-HRMS in positive ionization mode.

**Scheme 2:**
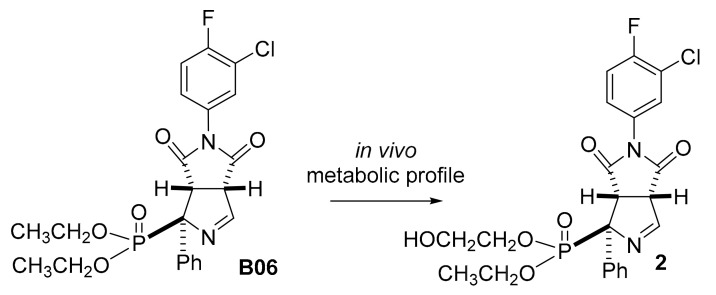
Putative oxidized product of **B06** resulting from the in vitro HLM assay.

In this condition, two potential metabolites of **B06**, 2-hydroxyethyl ((1R,3aS,6aS)-5-(3-chloro-4-fluorophenyl)-4,6-dioxo-1-phenyl-1,3a,4,5,6,6a-hexahydropyrrolo[3,4-c]pyrrol-1-yl)phosphonate, named as **2,** were tentatively identified in vivo from 30 to 480 min. The assigned molecular formula was C_22_H_21_N_2_O_6_FPCl, and taking into account the fragmentation patterns in the mass spectra, it seems that one of the oxidations occurred in one of the ethyl groups of the phosphonate ester, and the other oxidation could be predicted to be in the other ethyl group. These oxidations of Phase I accounted for the prochiral character of the phosphorous that when substituted by two ethoxy groups is lacking chirality. It is not a stereocentre, but when one of the substituents is altered, the phosphorous becomes prochiral; therefore, both ethoxy groups are different from the stereochemical point of view (Scheme 2).

The low concentration of metabolites made detection in plasma difficult due to the fact that concentrations of the parental **B06** molecule were also low. The potential metabolite of **B06** at 30 min was 2.5% of an oxidized **B06** derivative (*m*/*z* 495.0877), at 60 min was slightly increased to 2.8%, and at 120 min was detected in 5.8%. When increasing the time to 240 min, two tentative oxidation metabolites were found with slightly different retention times, 7.82 and 7.98 min, in the chromatogram in the conditions used. The new product at 7.98 min represented 1.5%, and the former oxidized metabolite (7.82 min) was 6.3%. At 360 min, only one of the oxidized **B06** proposed metabolite was detected at 6.7%, and at 480 min, the percentage was 4.5% in the order of the previous determinations.

**Figure 11:**
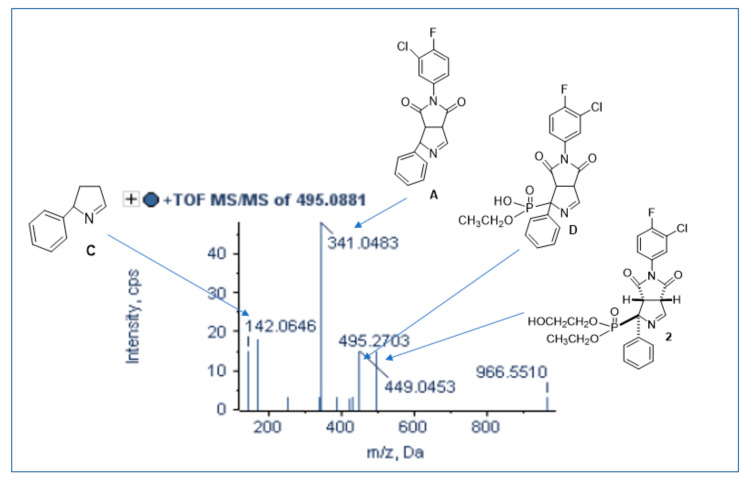
MS/MS spectra at 480 min of the characteristic fragmentation pattern of **B06** metabolite **2** in in vivo PK.

From the TOF MS/MS spectra at 480 min, several peaks were selected for assignment. The signal with the mass of the product **B06** with an additional 17 units, 495.2703, could represent the gain of an hydroxyl group. Other peaks selected for the assignment were 341.0498 that may belong to fragment **A** without the diethylphosphonate ester moiety of **B06**, 142.0646 that may correspond to fragment **C**, 2-phenyl-3,4-dihydro-2H-pyrrole, and 449.0453 that can be the mass of fragment **D** with loss of C_2_H moiety (Figure 11).

## 3. Materials and Methods

### 3.1. Synthesis of **B06**

To access enough quantities of **B06** for the studies reported in this paper, we undertook its synthesis by following a previously reported procedure [33]. The [3 + 2] cycloaddition reaction between diethyl α-phenylisocyanomethylphosphonate (187 mg, 0.7 mmol) and N-(4-chloro-4-fluorophenyl)maleimide (250 mg, 1.1 mmol) in acetonitrile (6 mL) and under AgOAc (8 mg, 0.05 mmol) catalysis furnished **B06** after purification by flash chromatography (189 mg, 54%). The relative stereochemistry of the three generated stereocentres was determined by X-ray crystallography. The representative data of ^1^H RMN, ^13^C RMN and HPLC-MS spectra are included in the Supplementary Materials (pages S19–S22) [33].

### 3.2. Solubility and Chemical Stability of **B06** at Different pH

The detection of **B06** at different pH (2, 5 and 7.4) was obtained by LC/MS/MS spectrometry with mobile phase A (0.1% formic acid in water/acetonitrile 90/10) and B (0.1% formic acid in acetonitrile/water 90/10) using an injection volume of 5 μL. The autosampler’s drawers were kept cooled at 4 °C. Diethyl (1*RS*,3a*SR*,6a*SR*)-5-phenyl-4,6-dioxo-1-phenyl-1,3a,4,5,6,6a-hexahydropyrrolo[3,4-*c*]pyrrole-1-phosphonate, an analogous molecule to **B06** without halogen substitution in the *N*-phenyl moiety, was used as the internal standard [33]. Detection of analytes and internal standards was carried out in multiple reaction monitoring mode (MRM) with electrospray positive ionization. Mass transitions used in the method were *m*/*z* 479.2/341.2 for **B06** (collision energy of 22 V) and *m*/*z* 427.2/289.3 for the internal standard (collision energy of 21 V). The mass spectrometric analysis can present a variability between 15–20% [43].

### 3.3. Plasma Stability of **B06** at Mouse Plasma

Mouse plasma pooled from healthy donors extracted in citrate tubes was employed in the assay. Plates containing 10 µM **B06** in plasma (total volume: 50 µL) were incubated at 37 °C at different times (0, 60, 180 and 360 min). Then, 100 µL of acetonitrile was added to precipitate the plasma protein, and the plate was centrifuged at 46,000× *g* for 60 min at 5 °C. Supernatant was taken and analysed by UPLC/MS/MS for sample quantification. The stationary phase was a reverse phase Acquity UPLC^®^BEH C18 1.7 µm (2.1 × 50 mm) (Waters), and the mobile phase was 0.1% formic acid in water/0.1% formic acid in acetonitrile, using a gradient and flow rate of 0.6 mL/min. The chromatographic equipment employed was an UPLC QSM Waters Acquity, and the compound concentrations were calculated from the MS peak areas.

### 3.4. Metabolic Stability of **B06** in Human Liver Microsomes

The methods and data related to this section are included in the Supplementary Materials (page S1) [44].

### 3.5. Metabolic Profiling of **B06** in Human and Mouse Liver Microsomes

The methods and data related to this section are included in the Supplementary Materials (page S3).

### 3.6. Chemical Synthesis

Ethyl hydrogen [(1R,3aS,6aS)-5-(3-chloro-4-fluorophenyl)-4,6-dioxo-1-phenyl-1,3a,4,5,6,6a-hexahydropyrrolo[3,4-c]pyrrol-1-yl]phosphonate (1).

To a solution of **B06** (100 mg, 0.21 mmol) in dichloromethane (5 mL), TMSBr (0.3 mL, 2.1 mmol) was added dropwise under argon. The mixture was stirred at room temperature for 1 h. The solvent was evaporated to afford **1**, which was purified by a reversed-phase column chromatography (H_2_O:ACN 95:5) [45]. ^1^H NMR (400 MHz, CD_3_OD) δ 1.17 (t, *J* = 7.0 Hz, 3H, CH_2_CH_3_), 3.87–4.13 (m, 3H, CH_2_CH_3_ and H-3a), 4.21 (dd, *J* = 4.5, 17.5 Hz, 1H, H-6a), 6.73 (dd, *J* = 8.0, 18.5 Hz, 2H, ArH), 7.19 (t, *J* = 9.0 Hz, 1H, ArH), 7.33 (d, *J* = 5.5 Hz, 3H, ArH), 7.66 (s, 2H, ArH), 7.97 (s, 1H, ArH), 8.20 (d, *J* = 7.5 Hz, 1H, H-3). HRMS C_20_H_16_ClFN_2_O_5_P [M + H]^−^ 449.0475; found, 449.0469. Purity 96.2% (t_R_ = 3.39 min). In addition, the compound [(1R,3aS,6aS)-5-(3-chloro-4-fluorophenyl)-4,6-dioxo-1-phenyl-1,3a,4,5,6,6a-hexahydropyrrolo[3,4-c]pyrrol-1-yl]phosphonic acid, resulting from the dihydrolysis reaction, was isolated: ^1^H NMR (400 MHz, CD_3_OD) δ 3.35 (s, 1H, H-3a), 4.22 (d, *J* = 17.0 Hz, 1H, H-6a), 6.73 (dd, *J* = 7.5, 18.0 Hz, 2H, ArH), 7.19 (t, *J* = 9.0 Hz, 1H, ArH), 7.32 (d, *J* = 6.5 Hz, 3H, ArH), 7.66 (s, 2H, ArH), 7.96 (s, 1H, H-3). HRMS C_18_H_12_ClFN_2_O_5_P [M + H]^−^ 421.0162; found, 421.0166. Purity 100% (t_R_ = 3.06 min).

The ^1^H RMN and HPLC-MS spectra are depicted in the Supplementary Materials (Pages S20–S21 and S23–S25).

### 3.7. In Vitro Study of the Role of **B06** in a Cellular Model of Neurodegeneration

#### 3.7.1. SH-SY5Y Human Cell Line

Cell lines of human neuroblastoma SH-SY5Y (Sigma-Aldrich, St. Louis, MO, USA) were propagated and maintained under standard conditions (37 °C and 5% CO_2_) in F12 medium/EMEM (Gibco, Waltham, MA, USA) containing 2 mM glutamine, 1% nonessential amino acids, and 15% fetal bovine serum (FBS). To test the excitotoxicity of the compounds, the cultures were treated for 24 h with the indicated compounds at 0.2, 0.5, 1, 2, 5, 10 and 20 μM. After that time, cell viability was assessed (Page S18, Figure S18) using the MTT assay. None of the concentrations used were cytotoxic to the cells. On attaining semiconfluence, cells were pretreated for 1 h with the different compounds at 1 μM. The dose was chosen based on a dose–response experiment previously performed in order to select the lowest concentration with the highest effect (Figure 7a). After that, 6-OHDA (25 μM, Sigma) was added to the cultures and incubated for 16 h. Finally, cultures were processed for cell viability assay and nitrite production.

#### 3.7.2. Primary Glial Cultures

Mouse cerebral cortex-derived glial cells were prepared as previously described [38]. Briefly, the brain was dissected and the cerebral cortex isolated, dissociated and incubated with 0.25% trypsin/EDTA at 37 °C for 1 h. Then, the tissue was centrifugated, and after several rinses in HBSS (Gibco), cells were plated on noncoated flasks and maintained in HAMS/DMEM (1:1) medium containing 10% FBS. After 7 days on culture, flasks were agitated on an orbital shaker for 4 h at 240 rpm at 37 °C, the supernatant was collected, centrifuged, and the cellular pellet containing the microglial cells resuspended in complete medium (HAMS/DMEM (1:1) containing 10% FBS) and seeded on uncoated 96-well plates. Cells were allowed to adhere for 2 h, and the medium was removed to eliminate nonadherent oligodendrocytes. New fresh medium containing 10 ng/mL of GM-CSF was added. The remaining astroglial cells adhered to the flasks were then trypsinized, collected, centrifuged, and plated onto 96-well plates with complete medium. The purity of cultures obtained by this procedure was >98% as determined by immunofluorescence with Iba-1 (microglial marker) and GFAP (astroglial marker) antibodies. After 2 days in culture, cells were pretreated for 1 h with the compounds (1 μM), and later with bacterial lipopolysaccharide (LPS; 10 μg/mL) for 24 h. Nitrite production on cultures was then measured.

#### 3.7.3. Cell Viability Assay and Apoptosis Measurement

Cell viability was measured using the MTT assay (Roche Diagnostic, GmbH, Basel, Switzerland), based on the ability of viable cells to reduce yellow MTT to blue formazan. Briefly, cells were cultured in 96-well plates and treated with the indicated compounds for 16 h, and then cells were incubated with MTT (0.5 mg/mL, 4 h) and subsequently solubilized in 10% SDS/0.01 M HCl for 12 h in the dark. The extent of reduction of MTT was quantified by absorbance measurement at 595 nm according to the manufacturer’s protocol. To evaluate the extent of apoptotic cell death, SH-SY5Y cultures were grown on glass cover-slips in 24-well cell culture plates for immunocytochemical analysis or in P60 plates for immunoblotting. After treatment, cover-slips were washed, permeabilized and treated as previously described [41]. A rabbit anti-active caspase-3 (1:200, MAB835 R&D Systems) primary antibody was used, followed by incubation with a 488-Alexa-labeled secondary antibody (Invitrogen, San Diego, CA, USA). Images were acquired using a Radiance 2100 confocal microscope (Bio-Rad, Hercules, CA, USA). Immunoblot analysis was performed as previously described [38]. Blots were probed with anti-human active caspase-3 (1:1000, MAB835 R&D Systems). Secondary peroxidase-conjugated donkey anti-rabbit antibody was purchased from Amersham Biosciences (GE Healthcare, Buckinghamshire, UK). Representative images of at least three independent experiments corresponding to three different samples are shown. The images of blotting were quantified using ImageJ software (Wayne Rasband, NIH, Bethesda, MD, USA). Values in the text are the mean of at least three different experiments.

#### 3.7.4. Nitrites Measurement

Accumulation of nitrites in the media was assayed by the standard Griess reaction. After stimulation of cells with the different treatments for 16 h, supernatants were collected and mixed with an equal volume of Griess reagent (Sigma-Aldrich). Samples were then incubated at room temperature for 15 min and absorbance read using a plate reader at 492/540 nm.

### 3.8. Method Validation for Quantification of **B06** in Mouse Plasma and Mouse Brain

The methods and data related to this section are included in the Supplementary Materials (Pages S8–S13, Tables S12–S19 and Figures S8–S13).

### 3.9. In Vivo Pharmacokinetics of **B06**

#### 3.9.1. Animals

Male CD1 mice were supplied by the Laboratory Animal Service of the University of Granada (Granada, Spain) and housed in the Animal Facilities of the Center of Biomedical Investigation of the University of Granada. They were housed in standard conditions (light/dark cycle of 12 h, temperature 22 ± 0.1 °C and 50–55% moisture) with free access to food (AIN-93G diet). Sample preparation: **B06** was dissolved in DMSO 10% and cyclodextrin 40% at 2 mg/mL. The calculated PK parameters for **B06** were obtained from all mouse plasma and brains. Calculations were performed using noncompartmental analysis of plasma data after intraperitoneal injection by means of PK Solver 2.0. All analytical determinations of **B06** were performed according to the recommendations of the FDA [46].

#### 3.9.2. Calibration Curve for **B06** in Plasma Samples

The methods and data related to this section are included in the Supplementary Materials (page S14, Figure S14).

#### 3.9.3. Quantitation for **B06** in Plasma Samples at Dose 10 mg/kg and Samples Grouping and Statistics in Plasma Samples

The methods and data related to this section are included in the Supplementary Materials (pages S14 and S15, Tables S20 and S21 and Figure S15).

#### 3.9.4. Calibration Curve for **B06** in Brain Samples

The methods and data related to this section are included in the Supplementary Materials (page S15, Figure S16).

#### 3.9.5. Quantitation for **B06** in Brain Samples at Dose 10 mg/kg and Samples Grouping and Statistics in Brain Samples

The methods and data related to this section are included in the Supplementary Materials (pages S16 and S17, Tables S22 and S23 and Figure S17).

### 3.10. Metabolic Profiling In Vivo of **B06**

The methods and data related to this section are included in the Supplementary Materials (pages S5 and S6).

### 3.11. Statistical Analysis

Figure 7 and Figure 8 are expressed as the mean ± SD of triplicate determinations. Experiments were repeated at least three times, yielding similar results. Data were analysed using one-way ANOVA. Then, a significance level of *p* < 0.05 was applied to the post hoc statistical analyses (Tukey test). The SPSS statistical software package (version 20.0) for Windows (Chicago, IL, USA) was used.

## 4. Conclusions

The discovery of **B06**, away from the structural limits of previous I_2_-IR ligands, has opened the door for the comprehensive exploration of these receptors in a plethora of indications. To start with, oral treatment with **B06** in murine models of neurodegeneration and AD prevented behavioural abnormalities and memory loss, reducing AD hallmarks, oxidative stress, and apoptotic and neuroinflammation markers. Preliminary ADME-Tox parameters and a safety panel proved that **B06** is a suitable compound to advance in preclinical phases.

In detail, in this work, we provided information on the metabolic stability of **B06** in human and mouse liver microsomes showing a medium hepatic clearance. The metabolic profile in liver microsomes lead to the proposal and unequivocal identification, after its synthesis, of a metabolite resulting from the monohydrolysis of the phosphonate ester function of the molecule. The in vivo PK study led to the calculation of relevant parameters and revealed a good correlation between the levels of **B06** found in plasma and in the brain, although the low amounts of the compound quantified indicated a good penetration in the CNS.

Notably, this paper is the first study aimed to unveil the role that I_2_-IR ligands could have in PD. After finding the optimal dose, the neuroprotective role of **B06** on the human dopaminergic cell line SH-SY5Y was determined. Furthermore, in primary cultures of astrocytes and microglia treated with LPS, **B06** was shown to be an anti-inflammatory agent even better than other known ligands.

Overall, our results confirm that **B06** is a promising candidate for neurodegenerative management due to its immunomodulatory and neuroprotective properties. **B06** is a robust compound to be seriously considered as a modulator of I_2_-IR for characterizing the pharmacological profile of these receptors and for further and more advanced preclinical development in brain disorders such as AD and PD.

## 5. Patents

The authors declare the following competing financial interest(s): C.E., M.P., and C.G.-F. are inventors of the patent application of I_2_ imidazoline receptor ligands, WO2019//121853 [32].

## Figures and Tables

**Table 1:** Percentage of **B06** remaining at pH 2 versus time (0–120 min).

Time (min)	% B06 Remaining
0	100 ± 8.84
5	104 ± 9.81
15	98 ± 11.30
30	106 ± 9.97
60	112 ± 11.2
120	96 ± 13.6

**Table 2:** Percentage of **B06** remaining at pH 5 versus time (0–120 min).

Time (min)	% B06 Remaining
0	100 ± 3.3
5	97 ± 6.5
15	100 ± 3.9
30	87 ± 11.3
60	98 ± 10.1
120	75 ± 8.5

**Table 3:** Percentage of **B06** remaining at pH 7.4 versus time (0–120 min).

Time (min)	% B06 Remaining
0	100 ± 2.9
15	77 ± 3.8
30	75 ± 5.6
60	53 ± 6.4
120	19 ± 1.9

**Table 4:** Percentage of **B06** remaining at each incubation time in HLM.

Time (min)	% Compound Remaining
	*Plus NADPH*
0	100.00 ± 14.46
5	98.60 ± 9.11
15	77.90 ± 13.20
30	54.10 ± 6.84
45	41.3 ± 5.83
60	29.40 ± 3.69
	*Minus NADPH*
0	103.2 ± 13.82
60	75.30 ± 1.27

**Table 5:** Percentage of **B06** remaining at each incubation time in MLM.

Time (min)	% Compound Remaining
	*Plus NADPH*
0	100.00 ± 25.58
5	135.00 ± 7.06
15	67.50 ± 4.15
30	30.30 ± 5.28
45	15.20 ± 1.98
60	6.08 ± 0.01
	*Minus NADPH*
0	105.00 ± 23.97
60	83.20 ± 2.87

## Data Availability

Not applicable.

## Supplementary Materials

The following supporting information can be downloaded at: https://www.mdpi.com/article/10.3390/ijms23105408/s1.

## Abbreviations

AD	Alzheimer’s disease
*α*_2_-AR	α_2_-adrenoceptors
BBB	blood−brain barrier
Cl	plasma clearance
C_max_	maximum observed concentration
HLM	human liver microsomes
I_2_-IR	imidazoline I_2_ receptors
LPS	bacterial lipopolysaccharide
MLM	mouse liver microsomes
MRM	reaction monitoring mode
ND	neurodegenerative diseases
6-OHDA	6-hydroxydopamine
PAMPA	parallel artificial membrane permeability assay
PD	Parkinson’s disease
PK	pharmacokinetics
SAMP8	senescence-accelerated mouse prone 8
