# Supplementary material for: Insights into the Pharmacokinetics and In Vitro Cell-Based Studies of the Imidazoline I2 Receptor Ligand B06
Source: Int J Mol Sci. 2022 May 12;23(10):5408. doi: 10.3390/ijms23105408 (PMC9141032; doi:10.3390/ijms23105408)

Insights into the pharmacokinetics and *in vitro* cell-based studies of the imidazoline I_2 receptor ligand B06.

Andrea Bagán[1], José A. Morales-García[2], Christian Griñán-Ferré[3], Caridad Díaz[4], José Pérez del Palacio[4], Maria C. Ramos [4], Francisca Vicente[4], Belén Pérez[5], José Brea[6], M. Isabel Loza[6], Mercè Pallàs[3], Carmen Escolano[1],*

1 Laboratory of Medicinal Chemistry (Associated unit to CSIC), Department of Pharmacology, Toxicology and Medicinal Chemistry, Faculty of Pharmacy and Food Sciences, and Institute of Biomedicine (IBUB), University of Barcelona, E-08028, Barcelona, Spain. abaganpolonio@ub.edu (A. B.); cescolano@ub.edu (C. E.).

2 Department of Cell Biology. School of Medicine. Complutense University (UCM), 28040 Madrid, Spain and The Network Center for Biomedical Research in Neurodegenerative Diseases (CIBERNED). jmoral06@ucm.es (J. A. M.).

3 Pharmacology Section, Toxicology and Medicinal Chemistry, Faculty of Pharmacy and Food Sciences, and Institut de Neurociències, University of Barcelona, E-08028 Barcelona, Spain. christian.grinan@ub.edu (C. G.-F.); pallas@ub.edu (M. P.).

4 Fundación MEDINA, Centro de Excelencia en Investigación de Medicamentos Innovadores en Andalucía, Avda. del Conocimiento 34, 18016 Armilla, Granada, Spain. caridad.diaz@medinaandalucia.es (C. D.); jose.perezdelpalacio@medinaandalucia.es (J. P.); carmen.ramos@medinaandalucia.es (M. C. R.); francisca.vicente@medinaandalucia.es (F. V.).

5 Department of Pharmacology, Therapeutic, and Toxicology, Autonomous University of Barcelona, E-08193, Barcelona, Spain. belen.perez@uab.cat (B. P.).

6 Innopharma screening platform. BioFarma research group. Centro de Investigación en Medicina Molecular y Enfermedades Crónicas (CIMUS). Universidad de Santiago de Compostela, Santiago de Compostela, Spain. pepo.brea@usc.es (J. B.); mabel.loza@usc.es (M.I. L.).

* Correspondence: cescolano@ub.edu

CONTENTS:

Metabolic stability of B06 in human liver microsomes ……………………………………. S1

Metabolic stability of B06 in mouse liver microsomes ……………………………………………… S3

Metabolic profiling of B06 in human liver microsomes …………………..…………..…… S5

Metabolic profiling of B06 in mouse liver microsomes ………………………………….…S6

Metabolic profiling of B06 *in vivo* ………………………………………………………… S7

Method validation for quantification of B06 in mouse plasma ……………………………. S8

Method validation for quantification of B06 in mouse brain ……………………………… S11

***In vivo* pharmacokinetics of B06 ………………………………………………………...... S14**

SH-SY5Y Human Cell Line …………………………………………………………………. S18

^{1}H and ^{13}C NMR spectra ……………………………………………………………………... S19

Representative data of ^{1}H-NMR spectra ……………………………………………………. S21

HPLC/MS analysis ……………………………………………………………………………. S22

Metabolic stability of B06 in human liver microsomes

B06 was dissolved in DMSO at 2 mg/mL. Assay conditions, total incubation volume 400 µL, **B06** concentration 1 µM, protein concentration 1 mg/mL, NADPH, 1.3 mM. Potassium phosphate buffer (100 mM) pH 7.4 and human liver microsomes, were purchased from Beckton Dickinson. Verapamil, $MgCl_2$ and NaDPH were purchased from Sigma-Aldrich, Germany. The equipment needed for the study was Beckman Coulter Refrigerated centrifuge, Agilent 1290 Infinity Liquid Chromatograph, CTC Analytics PAL HT-xt injector, Api4000 Mass Spectrometer. Total incubation volume 400 µL, **B06** concentration 1 µM, protein concentration 1 mg/mL, NADPH 1.3 mM.

Test compound: a stock solution of **B06** was prepared in DMSO at 25 mM. The final concentration in the assay was 1 µM. A stock solution of 2.66 mM NADPH was prepared by dissolving appropriate amount of NADPH in 100 mM potassium phosphate buffer.

Analysis: For the data analysis the area ratio of analyzed *versus* internal standard was used to calculate the percentage of remaning compound at each incubation time. The natural logarithm of the percentage of reamining **B06** was plotted versus incubation time to calculate the half-life using the following equation: Half-life ($T_{1/2}$) (min) = 0.693/*k* (the slope of the natural log of the percent remaining *versus* time). Intrinsic clearance was determined by the following equation: Cl_{int} H = Ln 2/$t_{1/2}$ (min) x volume incubation (mL)/microsomal protein (mg) x 45 (MPPGL) 1500 g human liver/ 70 Kg human body weight. Units for CL_{int} are usually expressed as mL/min/mg microsomal protein, MPPGL = referred to as mg microsomal protein per gram liver [45].

Table S1. Summary of LC-MS conditions

LC- MS Conditions	
HPLC	Agilent 1290
MS/MS	Api4000
Software	Analyst
Ionization Mode	Electrospray positive
Sample matrix	Microsomes+ NADPH + buffer
Column	Discovery HS C18, 2.1 X 5 mm, 3 µm
Mobile phase	[Water: Acetonitrile :0.1% Formic Acid]
Flow rate	0.45 mL/min
Source Temperature (ºC)	500
Column Temperature (º C)	20
Injection Volume (µL)	5
Run time (min)	5

Table S2. Percentage of verapamil remaining at each incubation time

	**Verapamil**
	Plus NADPH
Time (min)	% Compound remaining
0	100.00±5.20
5	79.3 ±6.29
15	45.6±3.49
30	19.7±2.47
45	10.4±1.48
60	0.62±0.51
	**Minus NADPH**
Time (min)	% Compound remaining
0	105.00±8.08
60	126.00±12.73

Table S3. Metabolic stability of B06 and verapamil in human liver

Compound	Intrinsic clearance (mL/min/mg protein)	$t_{1/2}$ (min)
Verapamil	53.1	12.6
B06	23.5	28.4

Table S4. Compound clearance category classification

Clearance Category	Intrinsic Clearance (µL/min/mg protein)
	Human
Low	< 8.6
Medium	8.6-47.0
High	> 47.0

Figure S1. Time course of metabolic stability of verapamil in human liver microsomes

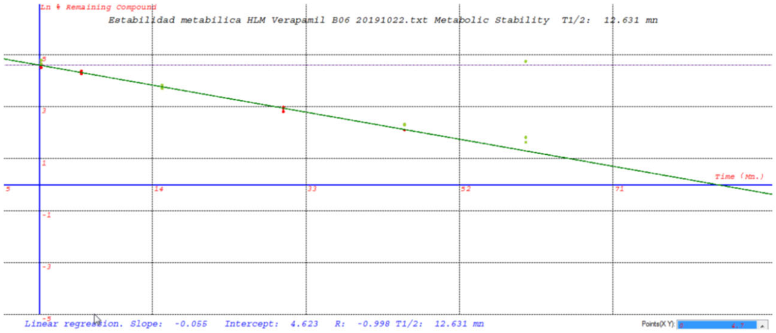

Figure S2.Time course of metabolic stability of B06 in human liver microsomes

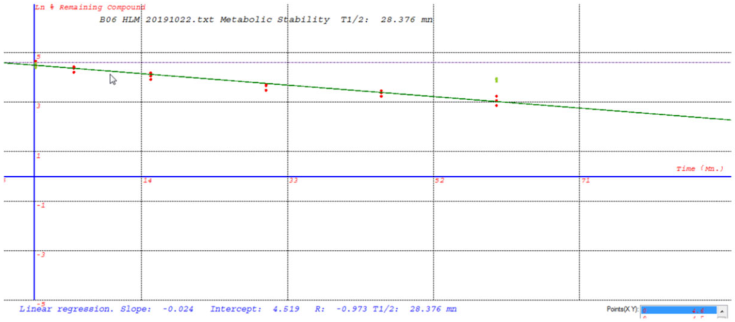

Metabolic stability of B06 in mouse liver microsomes

A stock solution of **B06** was prepared in DMSO at 2 mg/mL. The final concentration assay was 1 μM. A stock solution of 2.66 mM NADPH was prepared by dissolving appropriate amount of NADPH in 100 mM potassium phosphate buffer.

The equipment needed for the study was Agilent 1290 Infinity UHPLC, ABSciex 5600 QTOF Mass Spectrometer, Metabolite Pilot V 1.5 Software.

Method: Ionization mode, electrospray positive; adquisition mode, TOF MS with IDA MS/MS; column, Atlantis T3 C18, 2.1 x 15 mm, 3 μm; mobile phase, water: acetonitrile: 0.1%: formic acid; flow rate, 0.3 mL/min; source temperature, 550ºC; column temperature, 25ºC; injection volume, 5 μL, run time, 20 min. Total incubation volume 400 μL, **B06** concentration 1 μM, protein concentration 1 mg/mL, NADPH 1.3 mM.

Table S5. Summary of LC-MS conditions

LC- MS Conditions	
HPLC	Agilent 1290
MS/MS	Api4000
Software	Analyst
Ionization Mode	Electrospray positive
Sample matrix	Microsomes+ NADPH + buffer
Column	Discovery HS C18, 2.1 X 5 mm, 3 μm
Mobile phase	[Water: Acetonitrile :0.1% Formic Acid]
Flow rate	0.45 mL/min
Source Temperature (ºC)	500
Column Temperature (º C)	20
Injection Volume (μL)	5
Run time (min)	5

Table S6. Percentage of verapamil remaining at each incubation time

Time (min)	% compound remaining
	Plus NADPH
0	100.00 ± 0.40
45	0.08 ± 0.07
60	0.04 ±0.02
	Minus NADPH
0	78.40 ± 4.65
60	59.30 ± 2.63

Table S7. Metabolic stability of B06 and verapamil in mouse liver

Compound	Intrinsec clearance (mL/min/mg protein)	$t_{1/2}$ (min)
**Verapamil**	485.47	5.1
**B06**	153.7	16.23

$$CL_{int\ H} = \frac{Ln\ 2}{t_{\frac{1}{2}}(\mathrm{min})} * \frac{volume\ incubation\ (mL)}{microsomal\ protein\ (mg)} * 45(MPPGL)\frac{2\ g\ mouse\ liver}{0.025\ kg\ mouse\ body\ weight}$$

Table S8. Compound clearance category classification (mouse)

Clearance Category	Intrinsic Clearance (mL/min/mg protein)
	Mouse
Low	< 8.6
High	> 47.0

Figure S3. Time course of metabolic stability of verapamil in mouse liver microsomes

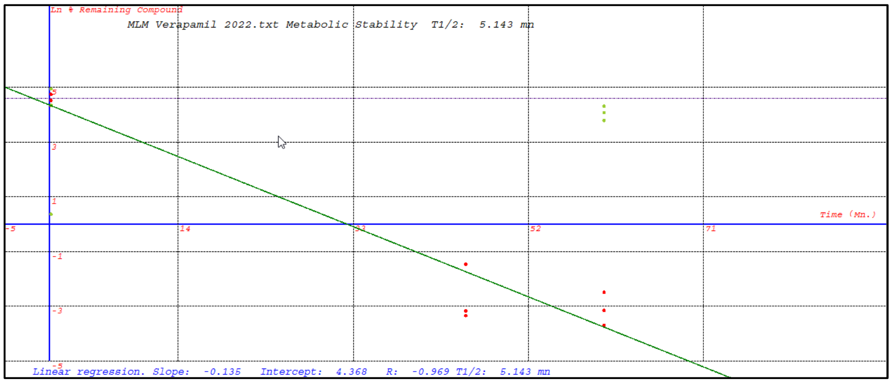

Figure S4. Time course of metabolic stability of B06 in mouse liver microsomes

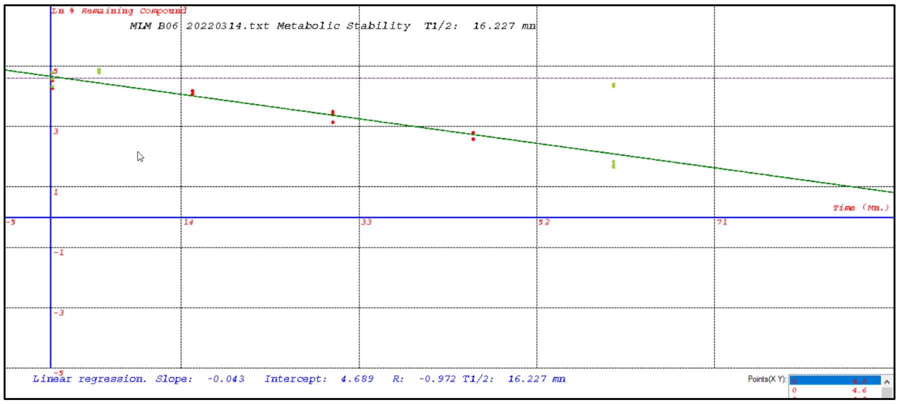

Metabolic profiling of B06 in human liver microsomes

The equipment used for the analysis was an Agilent 1290 Infinity UHPC, ABSciex 5600 QTOF Mass Spectrometer, Metabolite Pilot V 1.5 Sofware.

<u>Method</u>: ionization mode, electrospray positive; acquisition mode, TOF MS with IDA MS/MS; colum, Atlantis T3 C18, 2.1 x 15 mm, 3 μm; mobile phase, water:acetonitrile: 0.1% formic acid; flow rate, 0.3 mL/min; source temperature, 550ºC; column temperature, 25ºC; injection volume, 5 μL; run time, 20 min.

All the tentative identified metabolites have been evaluated using of a score (40 value). This score depend on mass defect, isotope pattern, mass accuracy and MS/MS. In all the cases the score was equal or greater than 68%.

<u>Table S9. Metabolite time course in HLM incubations for B06</u>

Peak ID	Description	Time incubation (min)	Formula	*m/z*	Mass error (ppm)	R.T. (min)	Peak Area	% Area	% Score
M1	Loss of C_2H_4	15	$C_{20}H_{17}N_2O_5FPCl$	451.0625	1.0	8.94	1.75E+04	8.4	81.1
M1	Oxidation	30	$C_{20}H_{17}N_2O_5FPCl$	451.0628	1.6	8.89	2.70e+04	17.8	75.0
M1	Loss of C_2H_4	45	$C_{20}H_{17}N_2O_5FPCl$	451.0624	0.8	8.97	3.70 E+04	26.2	85.5
M1	Loss of C_2H_4	60	$C_{20}H_{17}N2O5FPCl$	451.0624	0.9	8.90	4.03 E+04	36.0	81.8

<u>Figure S5. Metabolite time course in HLM incubations from 0 to 60 min for B06</u>

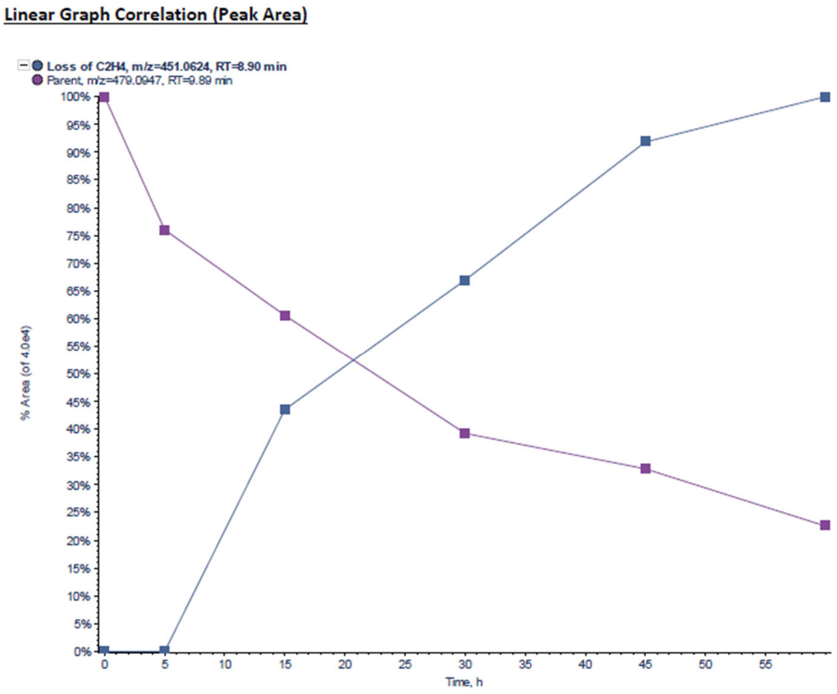

Metabolic profiling of B06 in mouse liver microsomes

The equipment used for the analysis was an Agilent 1290 Infinity UHPC, ABSciex 5600 QTOF Mass Spectrometer, Metabolite Pilot V 1.5 Sofware.

Method: ionization mode, electrospray positive; acquisition mode, TOF MS with IDA MS/MS; colum, Atlantis T3 C18, 2.1 x 15 mm, 3 µm; mobile phase, water:acetonitrile: 0.1% formic acid; flow rate, 0.3 mL/min; source temperature, 550ºC; column temperature, 25ºC; injection volume, 5 µL; run time, 20 min.

All the tentative identified metabolites have been evaluated using of a score (40 value). This score depend on mass defect, isotope pattern, mass accuracy and MS/MS. In all the cases the score was equal or greater than 68%.

All analytical determinations of **B06** were performed in according to the recommendations of the FDA.[1]

Table S10. Metabolite time course in MLM incubations for B06

Peak ID	Description	Time incubation (min)	Formula	*m/z*	Mass error (ppm)	R.T. (min)	Peak Area	% Area	% Score
M1	Loss of C_2H_4	5	$C_{20}H_{17}N_2O_5FPCl$	451.0620	0.0	8.88	2.74E+04	10.7	88.8
M1	Loss of C_2H_4	15	$C_{20}H_{17}N_2O_5FPCl$	451.0623	0.5	8.87	7.47E+04	30.9	90.8
M1	Loss of C_2H_4	30	$C_{20}H_{17}N_2O_5FPCl$	451.0617	-0.9	8.87	1.14E+05	60.9	92.0
M1	Loss of C_2H_4	45	$C_{20}H_{17}N_2O_5FPCl$	451.0623	0.6	8.83	1.06E+05	81.6	93.0
M1	Loss of C_2H_4	60	$C_{20}H_{17}N2O_5FPCl$	451.0620	-0.1	8.80	1.02E+05	88.9	92.5

Figure S6. Metabolite time course in MLM incubations from 0 to 60 min for B06

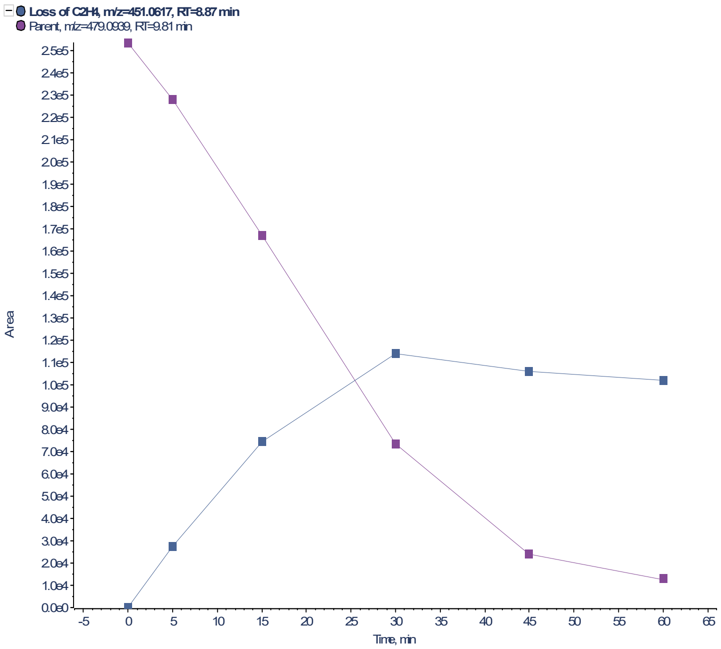

[1] Safety testing of drug metabolites. Guidance for industry. U.S. Department of Health and Human Services Food and Drug Administration Center for Drug Evaluation and Research (CDER) March 2020 Pharmacology/Toxicology. https://www.fda.gov/regulatory-information/search-fda-guidance-documents/safety-testing-drug-metabolites

Metabolic profiling of B06 *in vivo*

Table S11. Metabolite time course in vivo incubations for B06

Name	R.T. (min)	MS Area 30 min	MS Area 60 min	MS Area 120 min	MS Area 240 min	MS Area 360 min	MS Area 480 min
Oxidation 1	7.82	2.94E03	2.40E03	3.53E03	9.32E03	2.36E03	3.53E03
Oxidation 2	7.98	x	x	2.08E03	x	x	x
Parent	9.38	1.16E05	8.40E04	5.73E04	1.36E05	3.26E04	7.44E04

Figure S7. Metabolite time course in HLM incubations from 0 to 60 min for B06

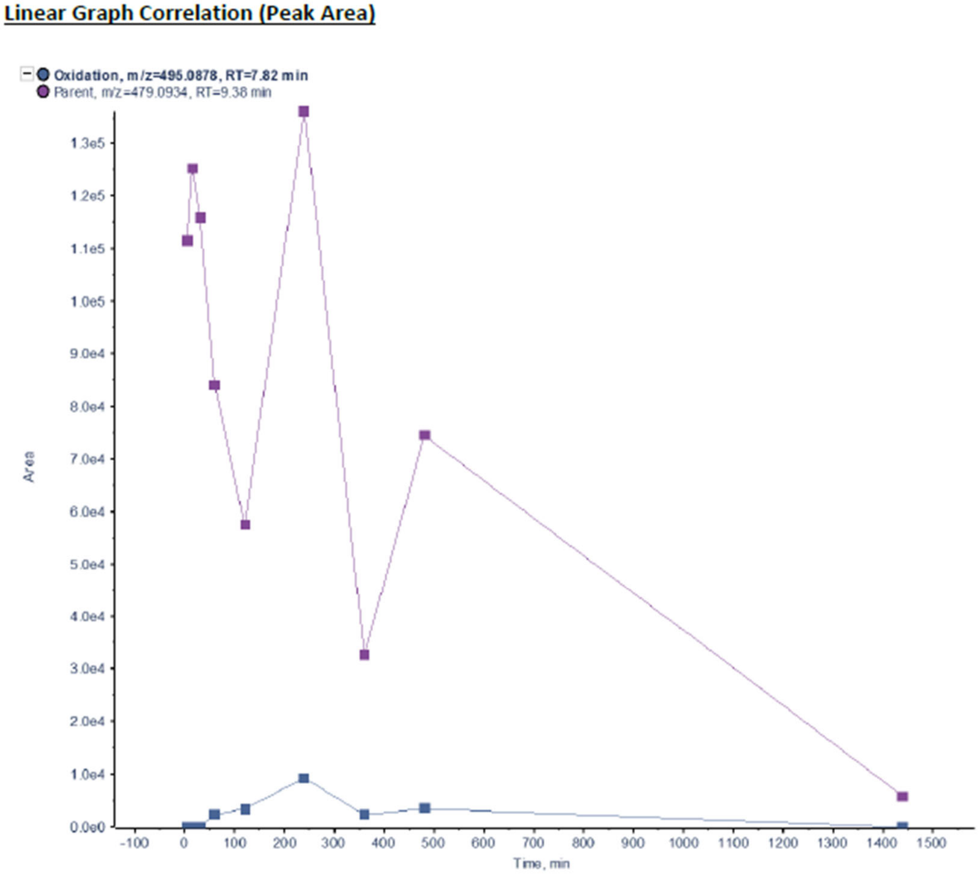

Method validation for quantification of B06 in mouse plasma

Table S12. Specificity of B06 and internal standard in mouse plasma samples

	Peak area at B06 RT in Blank	Peak area at B06 RT in LLOQ Sample	Blank Peak area at B06 RT (% of LLOQ)	Peak area at IS RT in Blank	Peak area at IS RT in LLOQ Sample	Blank Peak area at IS (% of LLOQ)
Blank 1	346	2310	14.98	11	277000	0.004
Blank 2	190	1640	11.59	15	268000	0.006
Blank 3	271	1540	17.60	13	264000	0.005
Blank 4	182	1640	11.10	10	261000	0.004
Blank 5	226	1690	13.37	17	243000	0.007
Blank 6	216	1480	14.59	13	265000	0.005
n	6	6	6	6	6	6
Mean	239	1717	13.9	13	263000	0.005

Table S13. Auto-sampler carry over test of B06 and internal standard in mouse plasma samples

Sample Name	Analyte	IS	% Carry Over	
			Analyte	IS
STD BLANK	347	70		
LLOQ	1880	339000		
ULOQ	264000	305000		
STD BLANK	328	171	17.4	0.6

Table S14. Back calculated values (ng/ml) data of cc standard for B06 in mouse plasma samples

Run Date	STD Conc. (ng/ml)	5.0	10.0	40.0	80.0	160.0	320.0	640.0
September	Run 1	4.9	10.9	38.4	77.0	148.8	308.5	670.0
	Run 2	5.2	10.9	40.1	81.8	141.0	322.4	649.5
Mean Calculated Concentration (ng/mL)		5.0	10.9	39.6	79.4	144.8	315.5	659.7
Accuracy (%)		100.8	108.6	99.1	99.3	90.5	98.6	103.1
Precision (CV %)		3.9	0.4	4.6	4.2	3.9	3.1	2.2
n		2	2	2	2	2	2	2

Table S15. Accuracy and precision of B06 in mouse plasma samples

Run Date	LQC (5.0 ng/mL)	MQC (71.1 ng/mL)	HQC (640.0 ng/mL)
September	6.7	71.3	666.1
	5.2	76.2	652.7
	5.0	70.7	638.5
	5.3	72.2	663.3
	5.7	68.9	609.1
	4.8	73.4	622.4
**Mean**	**5.4**	**72.1**	**642.0**
**Precision (CV %)**	**12.6**	**3.5**	**3.6**
**Accuracy (%)**	**108.7**	**101.6**	**100.3**
**n**	**6**	**6**	**6**

Figure S8. Blank sample chromatogram of B06 and internal standard in mouse plasma samples

A. Peak of B06

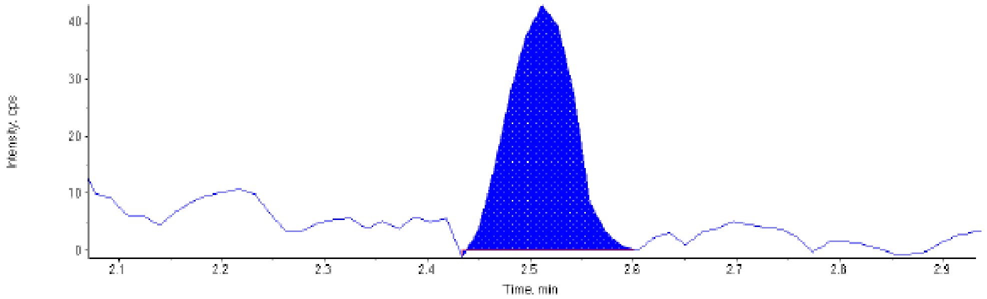

B. Peak of Internal standard

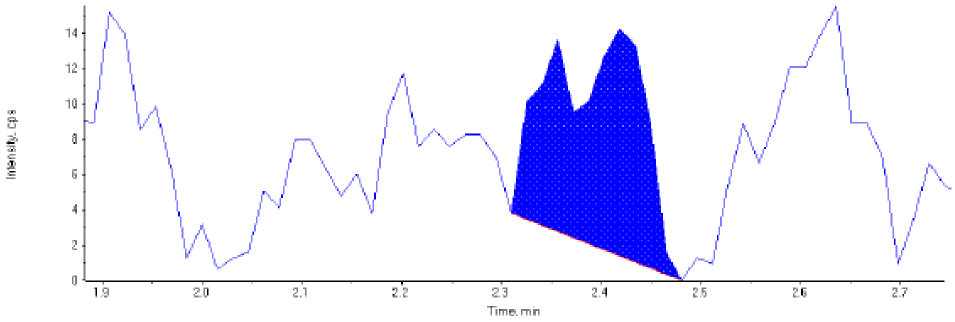

Figure S9. Chromatogram of B06 and internal standard in lloq sample in mouse plasma samples (concentration 5 ng/ml)

A. Peak of B06

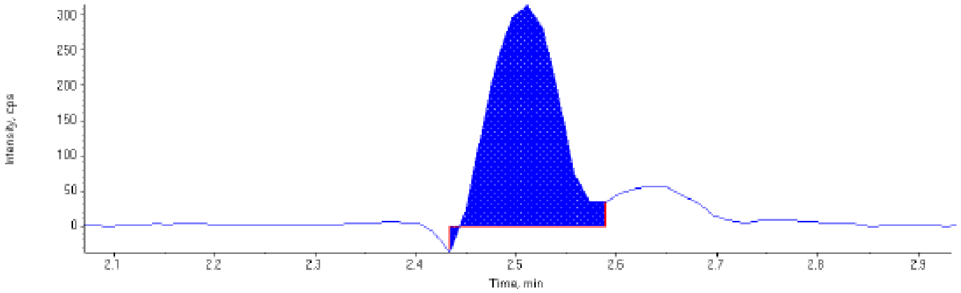

B. Peak of internal standard

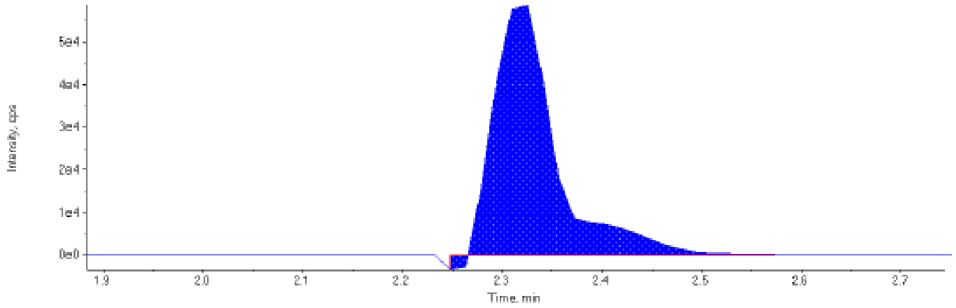

Figure S10. Standard curve of B06 in mouse plasma samples

Regression Equation Regression Equation: y = 0.00148 x + -0.00151 (r = 0.9988) (weighting: 1 / x)

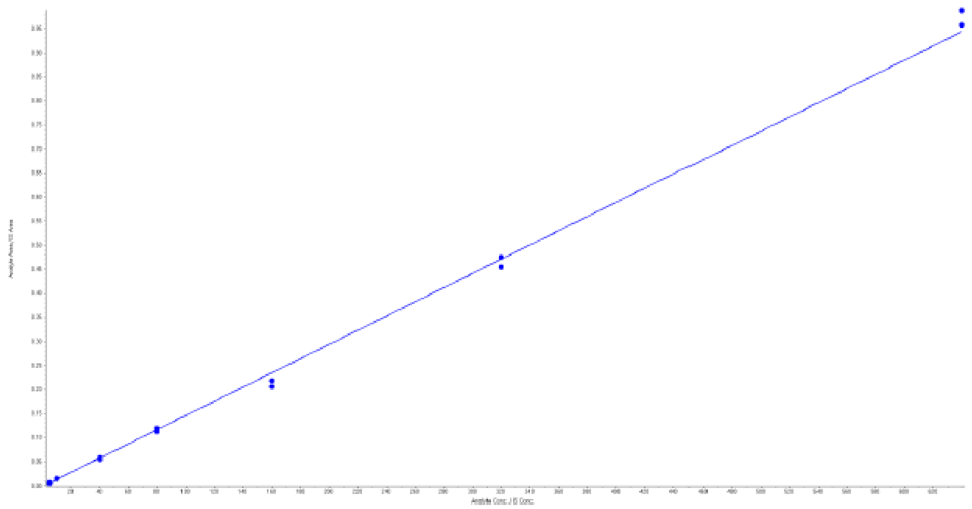

Method validation for quantification of B06 in mouse brain

Table S16. Specificity of B06 and internal standard in mouse brain samples

	Peak area at B06 RT in Blank	Peak area at B06 RT in LLOQ Sample	Blank Peak area at B06 RT (% of LLOQ)	Peak area at IS RT in Blank	Peak area at IS RT in LLOQ Sample	Blank Peak area at IS (% of LLOQ)
Blank 1	133.0	1610	8.3	8.3	312000	0.003
Blank 2	90.5	1560	5.8	24.8	339000	0.007
Blank 3	45.6	1190	3.8	10.3	206000	0.005
Blank 4	116.0	1330	8.7	35.1	202000	0.017
Blank 5	58.9	1440	4.1	16.7	215000	0.008
Blank 6	54.0	1470	3.7	22.0	297000	0.007
n	6	6	6	6	6	6
Mean	83	1433	5.7	19.5	261000	0.008

Table S17. Auto-sampler carry over test of B06 and internal standard in mouse brain samples

Sample Name	Analyte	IS	% Carry Over	
			Analyte	IS
STD BLANK	106	62		
LLOQ	1350	279000		
ULOQ	332000	224000		
STD BLANK	176	78	13.0	0.03

Table S18. Back calculated values (ng/ml) data of cc standard for B06 in mouse brain samples

Run Date	STD Conc. (ng/ml)	2.5	5.0	10.0	20.0	40.0	80.0	160.0	320.0	640.0
September	Run 1	2.7	4.9	9.7	18.4	37.7	75.7	158.8	326.1	654.5
	Run 2	3.0	4.5	8.2	18.8	36.9	79.1	150.9	328.2	636.5
Mean Calculated Concentration (ng/mL)		2.9	4.7	9.0	18.6	37.3	77.4	154.8	327.2	645.5
Accuracy (%)		114.1	94.1	89.7	92.9	93.2	96.7	96.8	102.2	100.9
Precision (CV %)		7.9	7.3	12.3	1.6	1.5	3.1	3.6	0.4	2.0
n		2	2	2	2	2	2	2	2	2

Table S19. Accuracy and precision of B06 in mouse brain samples

Run Date	LQC (5.0 ng/mL)	MQC (71.1 ng/mL)	HQC (640.0 ng/mL)
September	5.3	65.0	611.6
	5.8	66.7	601.6
	5.8	65.8	606.0
	5.1	64.6	622.6
	5.4	61.9	624.7
	5.0	64.2	581.6
**Mean**	**5.4**	**64.7**	**608.0**
**Precision (CV %)**	**6.5**	**2.5**	**2.6**
**Accuracy (%)**	**108.2**	**91.1**	**95.0**
**n**	**6**	**6**	**6**

Figure S11. Blank sample chromatogram of B06 and internal standard in mouse brain samples

A. Peak of B06

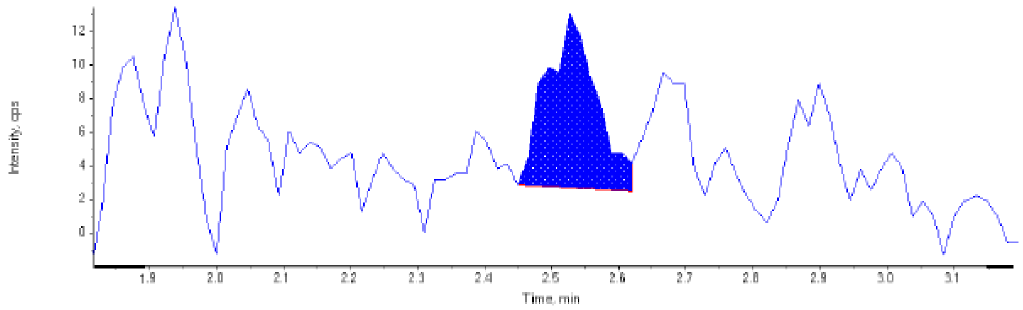

B. Peak of Internal standard

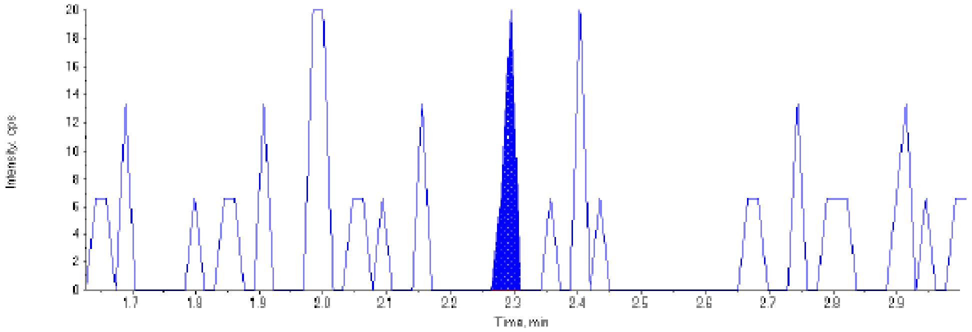

Figure S12. Chromatogram of B06 and internal standard in LLOQ sample in mouse brain samples (concentration 2.5 ng/ml)

A. Peak of B06

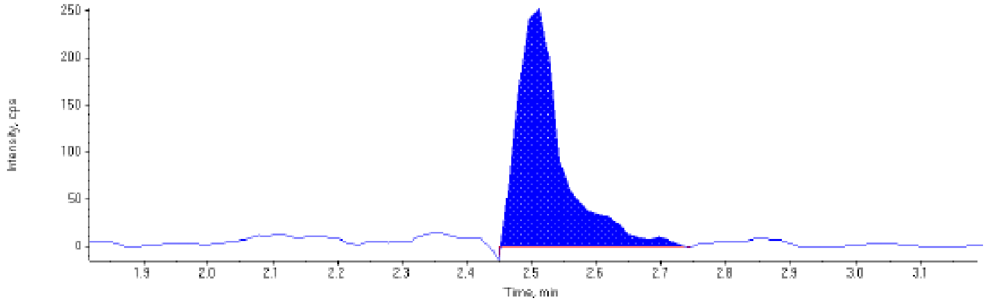

B. Peak of internal standard

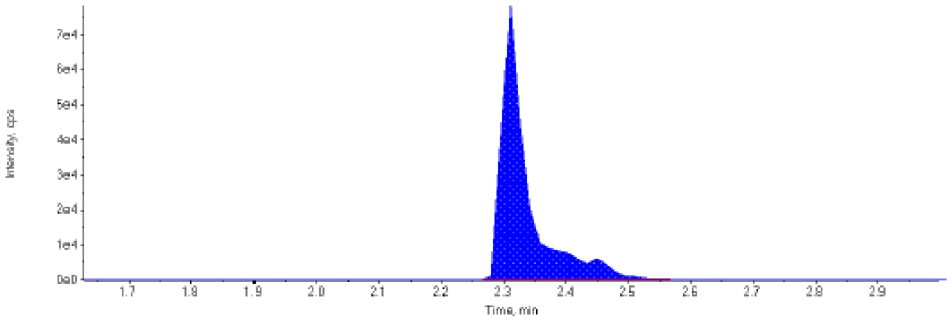

Figure S13. Standard curve of B06 in mouse brain samples

Regression Equation Regression Equation: y = 0.00253 x + -0.00103 (r = 0.9994) (weighting: 1 / x)

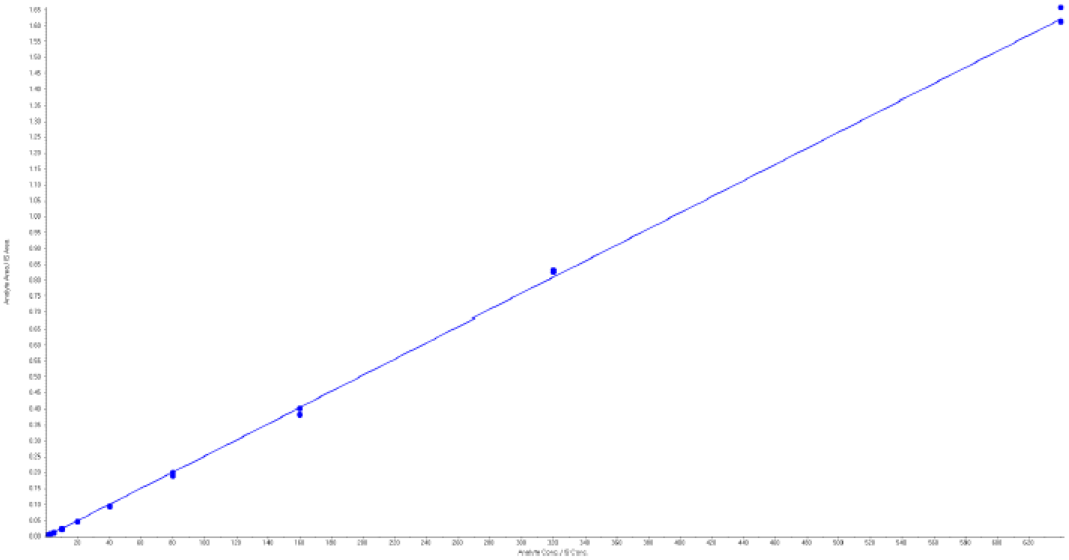

In vivo pharmacokinetics of B06

Figure S14. Calibration curve for B06 in plasma samples

Regression Equation: y = 0.00119 x + 0.000928 (r = 0.9993) 1/x

Expected Concentration	Number of Values	Mean Calculated Concentration	% Accuracy	Std. Deviation	%CV
5	2	4.46	89.2	0.01	0.1
10	2	10.69	106.9	0.16	1.5
20	2	20.71	103.5	1.03	5.0
40	2	39.52	98.8	0.14	0.3
80	2	80.13	100.2	9.76	12.2
160	2	167.83	104.9	6.71	4.0
320	3	310.72	97.1	9.73	3.1
640	3	643.73	100.6	13.41	2.1

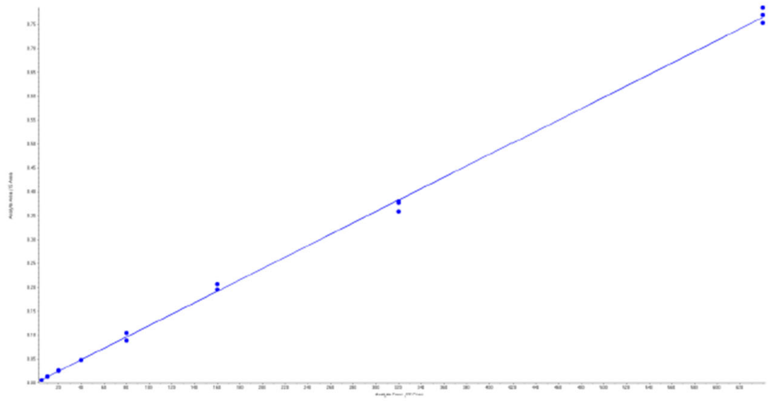

Table S20. Quantitation for B06 in plasma samples at dosing 10 mg/kg and samples grouping and statistics in plasma samples

Sample Name	Time (minutes)	10 mg/Kg Final. Conc. (ng/mL)
1	5	603
2	5	79
3	5	317
4	15	1580
5	15	332
6	15	939
7	30	263
8	30	293
9	30	320
10	60	577
11	60	125
12	60	185
13	120	138
14	120	225
15	120	391
16	240	< 5
17	240	123
18	240	315
19	360	90
20	360	146
21	360	96.6
22	480	81.8
23	480	156
24	480	174
25	1440	11
26	1440	14
27	1440	15

Table S21. Samples Grouping and statistics in plasma samples

N	Extraction_time (minutes)	Mean (ng/mL)	SD (ng/mL)	CV%
3	5	333.0	262.4	78.8
3	15	950.3	624.1	65.7
3	30	292.0	28.5	9.8
3	60	295.7	245.5	83.0
3	120	251.3	128.5	51.1
3	240	219.0	135.8	62.0
3	360	110.9	30.6	27.6
3	480	137.3	48.9	35.6
3	1440	13.3	2.1	15.6

Figure S15. B06 concentration in plasma samples vs. extraction time

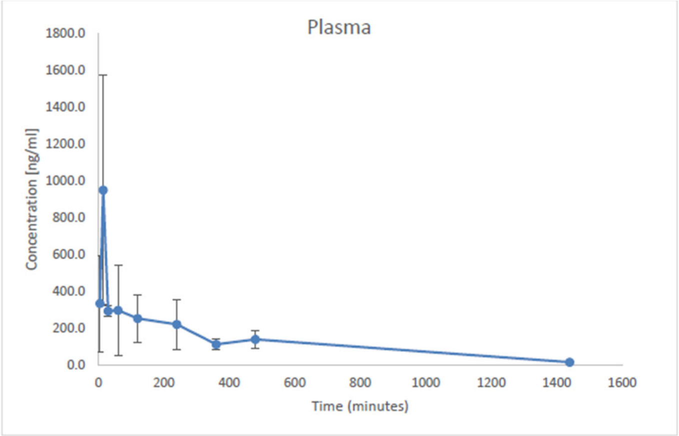

Figure S16. Calibration curve for B06 in brain samples

Regression Equation: y = 0.00212 x + 0.00082 (r = 0.9992) 1/x

Expected Concentration	Number of Values	Mean Calculated Concentration	% Accuracy	Std. Deviation	%CV
2.5	2	2.5	101.0	0.2	9.1
5	2	5.2	103.8	0.5	9.3
10	2	10.4	104.2	0.4	3.8
20	2	20.1	100.4	1.1	5.3
40	2	40.6	101.6	4.1	10.0
80	2	83.0	103.7	6.0	7.2
160	2	156.5	97.8	7.3	4.7
320	2	322.5	100.8	24.8	7.7
640	2	636.9	99.5	3.4	0.5

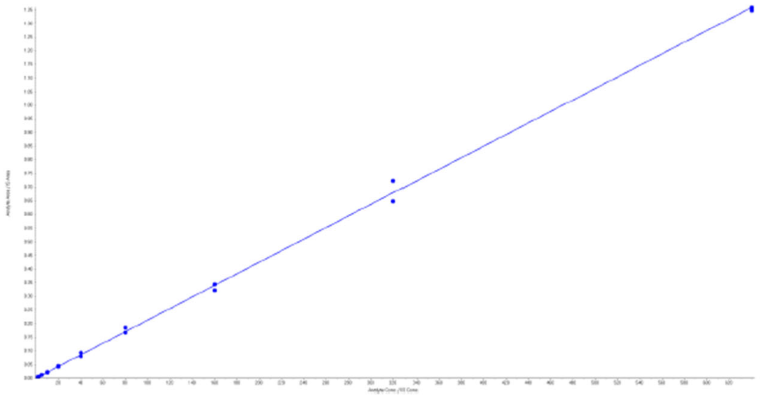

Table S22. Quantitation for B06 in brain samples at dosing 10 mg/kg and samples grouping and statistics in brain samples

B06	TIME (minutes)	CONCENTRATION (ng/ml)	CONCENTRATION (ng/g tissue)
1	5	17.2	60.2
2	5	3.2	11.2
3	5	12.5	43.75
4	15	51.8	181.3
5	15	12.2	42.7
6	15	46.1	161.35
7	30	15.0	52.5
8	30	14.1	49.35
9	30	14.7	51.45
10	60	19.4	67.9
11	60	4.4	15.4
12	60	8.71	30.485
13	120	6.62	23.17
14	120	6.95	24.325
15	120	10.8	37.8
16	240	< 2.5	N.d.
17	240	4.9	17.15
18	240	15.5	54.25
19	360	< 2.5	N.d.
20	360	3.96	13.86
21	360	2.69	9.415
22	480	< 2.5	N.d.
23	480	4.46	15.6
24	480	4.68	16.4
25	1440	< 2.5	N.d.
26	1440	< 2.5	N.d.
27	1440	< 2.5	N.d.

N.d. = Non-determinated

Table S23. Samples gruoping and statistics in brain samples

N	Extraction_time (hours)	Mean (ng/g tissue)	SD (ng/g tissue)	CV%
3	5	38.4	24.9	65.0
3	15	128.5	74.9	58.3
3	30	51.1	1.6	3.1
3	60	37.9	27.0	71.3
3	120	28.4	8.1	28.6
2	240	35.7	26.2	73.5
2	360	11.6	3.1	27.0
2	480	16.0	0.6	3.5

Figure S17. B06 concentration in brain samples vs. extration time

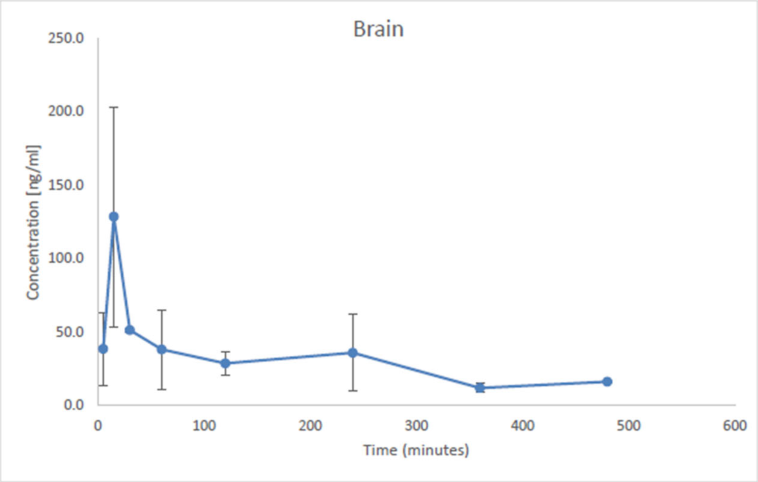

SH-SY5Y Human Cell Line

Figure S18. Study of the cytotoxic effect of I2-IR ligands on human dopaminergic cells

Cell line SH-SY5Y was treated for 24h with compounds B06, Idazoxan and 2-BFI at different concentrations from 0.2 to 20 uM. The cytotoxic effect of the compounds was analyzed by MTT assay. Values represent the mean ± SD from triplicate determinations repeated at least three times.

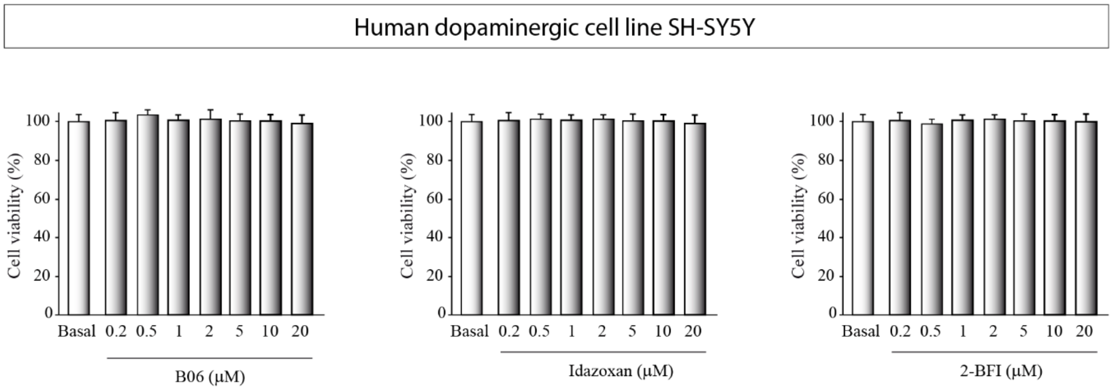

^{1}H and ^{13}C NMR spectra

Diethyl (1RS,3aSR,6aSR)-5-(3-chloro-4-fluorophenyl)-4,6-dioxo-1-phenyl-1,3a,4,5,6,6a-hexahydropyrrolo[3,4-*c*]pyrrole-1-phosphonate (B06)

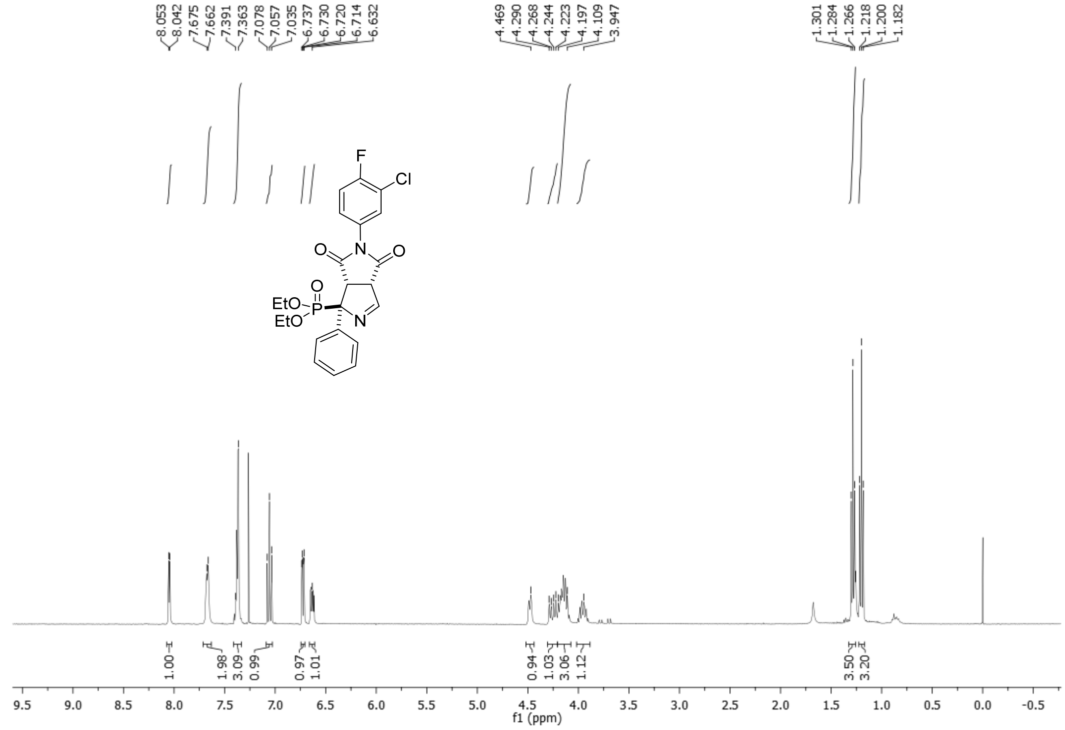

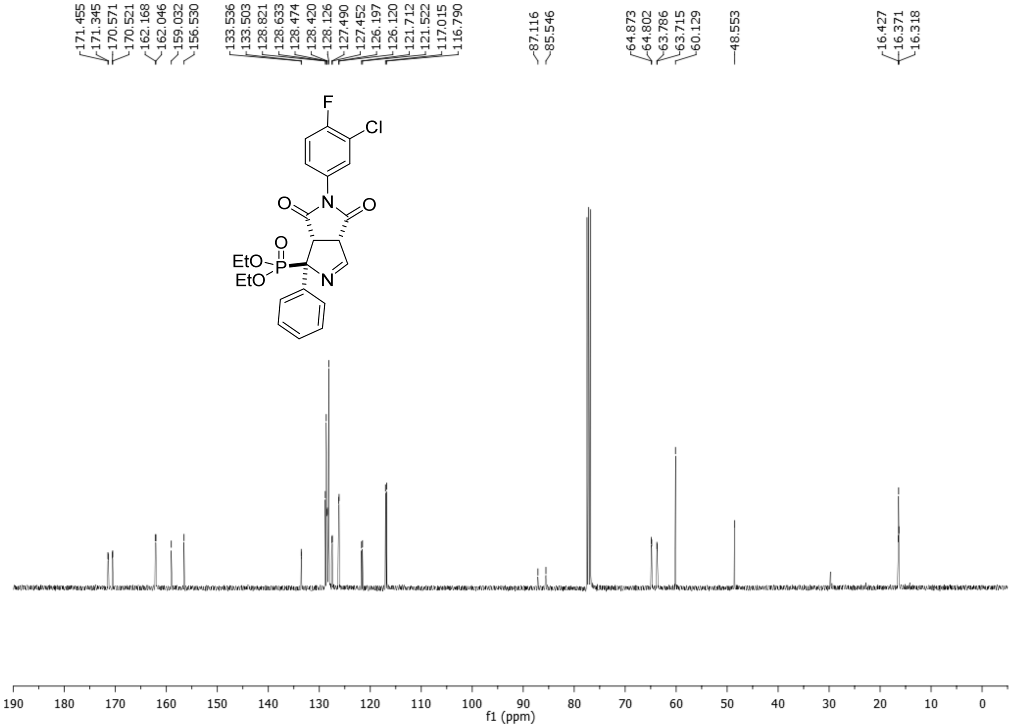

Ethyl hydrogen ((1R,3aS,6aS)-5-(3-chloro-4-fluorophenyl)-4,6-dioxo-1-phenyl-1,3a,4,5,6,6a-hexahydropyrrolo[3,4-*c*]pyrrol-1-yl)phosphonate (1)

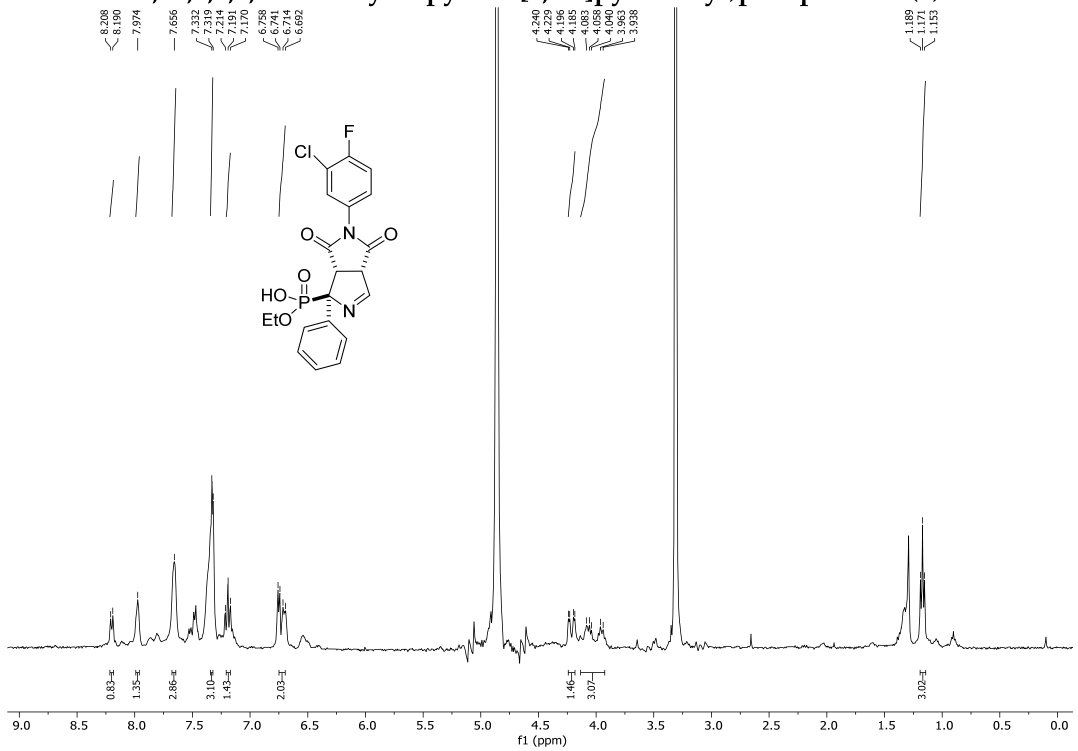

((1R,3aS,6aS)-5-(3-chloro-4-fluorophenyl)-4,6-dioxo-1-phenyl-1,3a,4,5,6,6a-hexahydropyrrolo[3,4-*c*]pyrrol-1-yl)phosphonic acid

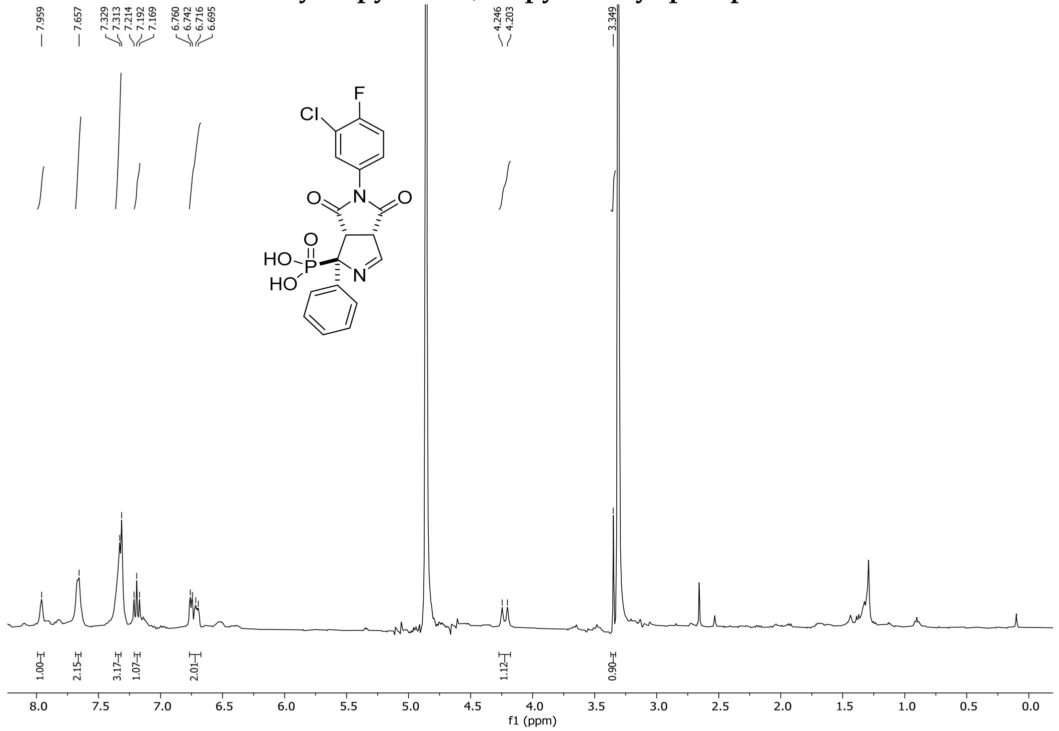

Representative data of ^{1}H-NMR spectra

Table S24. [1]H chemical shifts for new compounds including both the multiplicity and the coupling constants

Compound	CH_2CH_3	CH_2CH_3	H3	H6a	H3a
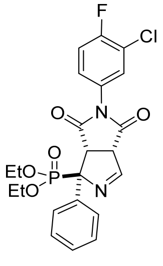 **B06**	1.20<br>t<br>7.0	1.28<br>t<br>7.0	8.05<br>d<br>4.5	4.25<br>dd<br>8.0, 18.0	4.47<br>m
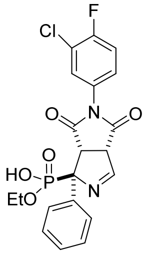 **1**	1.17<br>t<br>7.0	-	8.20<br>d<br>7.5	4.21<br>dd<br>4.5, 17.5	3.87-4.13<br>m
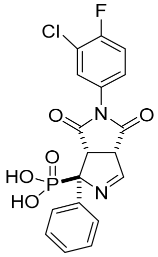	-	-	7.96<br>s	4.22<br>d<br>17.0	3.35<br>s

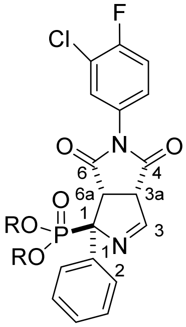

HPLC/MS analysis

Diethyl (1RS,3aSR,6aSR)-5-(3-chloro-4-fluorophenyl)-4,6-dioxo-1-phenyl-1,3a,4,5,6,6a-hexahydropyrrolo[3,4-*c*]pyrrole-1-phosphonate (B06)

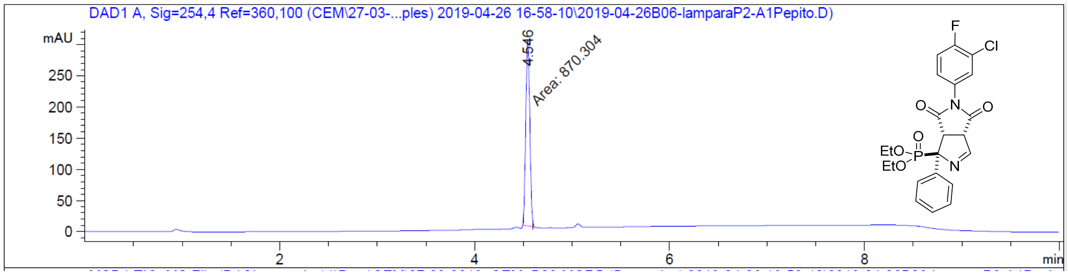

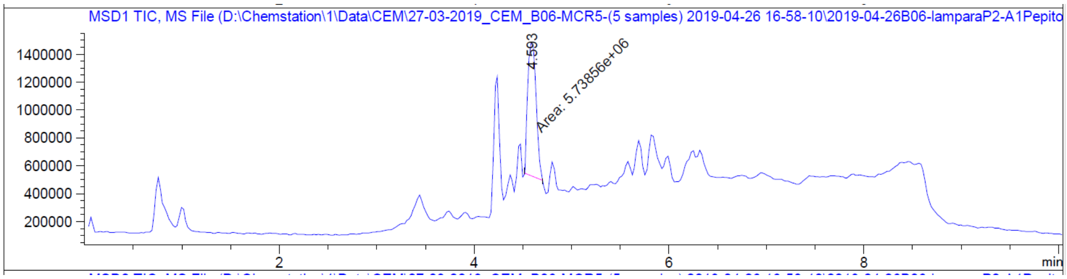

Signal 1: DAD1 A, Sig=254,4 Ref=360,100

Peak #	RetTime [min]	Type	Width [min]	Area [mAU*s]	Height [mAU]	Area %
1	4.546	MM	0.0483	870.30444	300.38943	100.0000
Totals :				870.30444	300.38943	

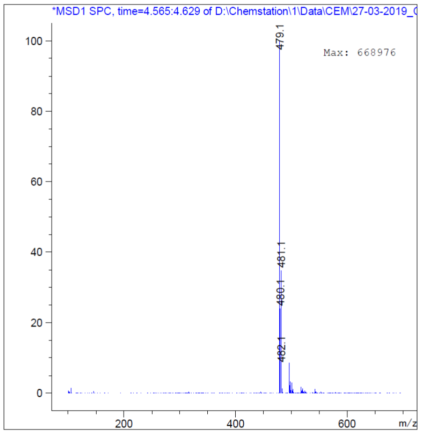

Ethyl hydrogen ((1R,3aS,6aS)-5-(3-chloro-4-fluorophenyl)-4,6-dioxo-1-phenyl-1,3a,4,5,6,6a-hexahydropyrrolo[3,4-*c*]pyrrol-1-yl)phosphonate (1)

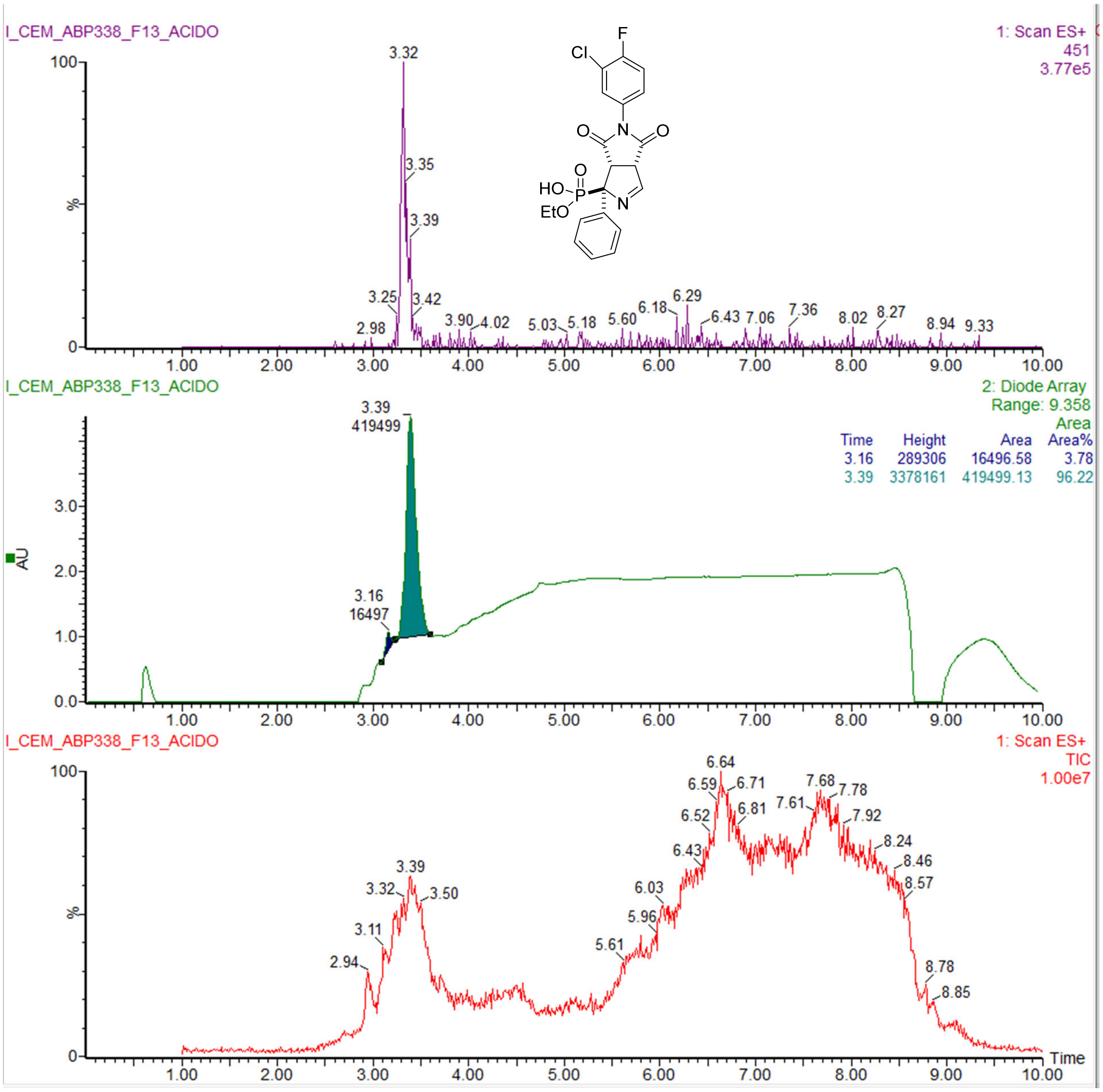

((1R,3aS,6aS)-5-(3-chloro-4-fluorophenyl)-4,6-dioxo-1-phenyl-1,3a,4,5,6,6a-hexahydropyrrolo[3,4-*c*]pyrrol-1-yl)phosphonic acid

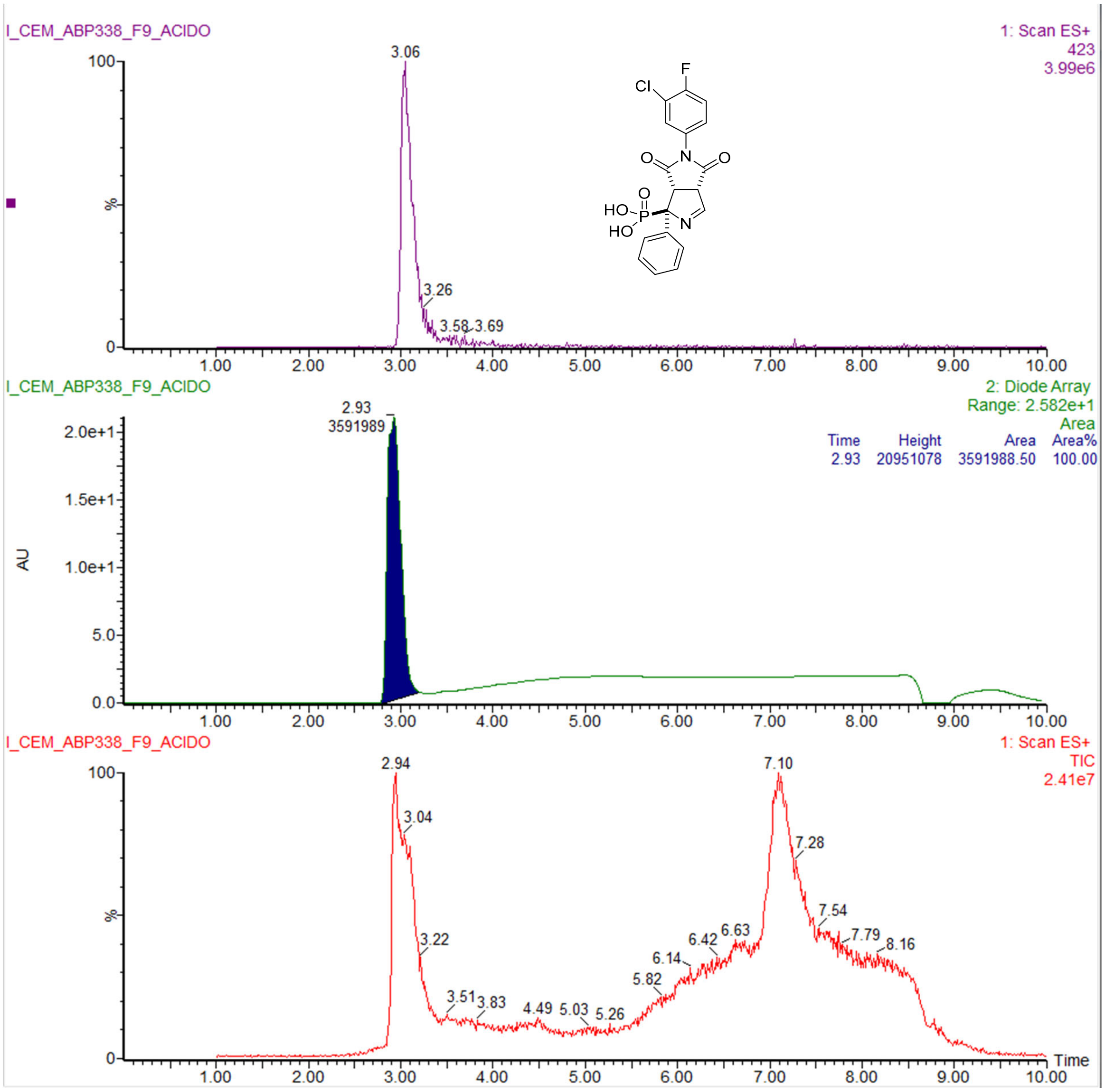

Supplement: Supplementary file 1 [file ijms-23-05408-s001.zip › ijms-1667157-supplementary.pdf]
